# Expression Atlas of the Deubiquitinating Enzymes in the Adult Mouse Retina, Their Evolutionary Diversification and Phenotypic Roles

**DOI:** 10.1371/journal.pone.0150364

**Published:** 2016-03-02

**Authors:** Mariona Esquerdo, Xavier Grau-Bové, Alejandro Garanto, Vasileios Toulis, Sílvia Garcia-Monclús, Erica Millo, Ma José López-Iniesta, Víctor Abad-Morales, Iñaki Ruiz-Trillo, Gemma Marfany

**Affiliations:** 1 Departament de Genètica, Facultat de Biologia, Universitat de Barcelona, Barcelona, Spain; 2 Institut de Biologia Evolutiva (CSIC- Universitat Pompeu Fabra), Barcelona, Spain; 3 Centro de Investigación Biomédica en Red de Enfermedades Raras (CIBERER), Instituto de Salud Carlos III, Barcelona, Spain; 4 Institució Catalana de Recerca i Estudis Avançats (ICREA), Barcelona, Spain; 5 Institut de Biomedicina de la Universitat de Barcelona (IBUB), Barcelona, Spain; University of Florida, UNITED STATES

## Abstract

Ubiquitination is a relevant cell regulatory mechanism to determine protein fate and function. Most data has focused on the role of ubiquitin as a tag molecule to target substrates to proteasome degradation, and on its impact in the control of cell cycle, protein homeostasis and cancer. Only recently, systematic assays have pointed to the relevance of the ubiquitin pathway in the development and differentiation of tissues and organs, and its implication in hereditary diseases. Moreover, although the activity and composition of ubiquitin ligases has been largely addressed, the role of the deubiquitinating enzymes (DUBs) in specific tissues, such as the retina, remains mainly unknown. In this work, we undertook a systematic analysis of the transcriptional levels of DUB genes in the adult mouse retina by RT-qPCR and analyzed the expression pattern by *in situ* hybridization and fluorescent immunohistochemistry, thus providing a unique spatial reference map of retinal DUB expression. We also performed a systematic phylogenetic analysis to understand the origin and the presence/absence of DUB genes in the genomes of diverse animal taxa that represent most of the known animal diversity. The expression landscape obtained supports the potential subfunctionalization of paralogs in those families that expanded in vertebrates. Overall, our results constitute a reference framework for further characterization of the DUB roles in the retina and suggest new candidates for inherited retinal disorders.

## Introduction

Ubiquitination is a dynamic regulatory mechanism that controls cell processes such as protein quality control (via proteasome degradation), cellular signalling, transcriptional regulation or DNA repair [1–3]. As ubiquitination is reversible, cells deploy a large set of enzymes to conjugate (E1, E2 and E3 ligases) and deconjugate (deubiquitinating enzymes) ubiquitin moieties [4]. The human genome contains several hundreds of ubiquitin ligases, and close to 80 deubiquitinating enzymes (DUBs), indicating that: i) ubiquitination is a highly regulated process, and ii) substrate recognition specificity is inherent to the system.

Most data on the physiological relevance of ubiquitin has focused on its role as the tag molecule to target substrates to proteasome degradation, its role in cell cycle control and cancer, as well as its involvement in the molecular basis of neurodegenerative disorders [5,6]. Besides, a number of high-throughput approaches have focused on finding substrates for either ligases [7] or deubiquitinating enzymes (DUBs) [8]. Nonetheless, most high-throughput studies have been performed *in vitro* using mammalian cell cultures, and only recently, systematic assays in animal models have indicated the relevance of the ubiquitin pathway in the development, differentiation and maintenance of tissues and organs [9,10].

One of the tissues that requires a tight gene and protein regulation is the retina. The retina consists of structured layers of highly specialized neurons in the eye that capture and process light stimuli enabling vision [11]. Such a fine architecture turns retinal differentiation into an extremely complex mechanism that must be accurately regulated [12], and in which ubiquitin and ubiquitination play a relevant role. In fact, mutations in the genes encoding the E3 ligases TOPORS [13–15] and KLHL7 [16,17]; and in PRPF8, which belongs to the JAB1-MPN-MOV34 (JAMM) family of DUBs, are causative of the most prevalent retinal hereditary dystrophy, retinitis pigmentosa (RP). Moreover, protein homeostasis via the ubiquitin-proteasome system is also relevant to other retinal diseases and specific altered protein degradation has been associated to Stargardt's disease, age-related macular degeneration, glaucoma, diabetic retinopathy, and retinal inflammation (reviewed in [18]).

Lately, DUBs are becoming the focus of attention given that their specificity in substrate selection makes them key checkpoints of protein degradation and fate. Moreover, their fewer numbers (compared to E2 and E3 ligases) makes their functional analysis more feasible. An increasing number of reports propose DUBs as pharmacological targets in disease: cancer [19–21] and neurodegenerative diseases [6]. DUBs are classified into five different subfamilies depending on their catalytic domains [22]: Machado-Joseph Disease protein domain proteases (MJD), Ovarian Tumor proteases (OTU), Ubiquitin C-Terminal Hydrolases (UCH) and Ubiquitin-Specific Proteases (USP) are cysteine proteases, whereas JAB1/MPN/MOV34 family proteases (JAMM) are Zn^2+^ metalloproteases; overall adding up to 90 genes in the human genome, of which only 79 are predicted to be functional [1].

A recent review compiled the gathered knowledge of the functional roles of individual DUBs, focusing on their subcellular localization, levels of expression in human tissues, and gene mutation phenotype in human and model organisms [23], yet a comprehensive study on the expression pattern of DUBs in highly specialized tissues, such as the retina, has not been performed. Besides, previous comparisons of DUB mutant phenotypes in different model organisms attempt to directly assign, without a phylogenetic framework, orthology and function between invertebrate and vertebrate genes. Some of these assignments may need revision under robust phylogenetic data, since ubiquitin ligase and protease families have expanded in eukaryotes [24], and subfunctionalization and neofunctionalization are known to occur after gene expansion.

Thus, we here aimed to draw an expression pattern map for DUB genes in the mouse retina, by using RT-qPCR, *in situ* hybridization and immunohistochemistry. We have also applied comparative genomics to infer the basic protein domain architecture within the DUB subfamilies and illustrate their diversification within metazoans. These data combined with the reported phenotypes will help to identify relevant retinal genes and potential new candidates for retinal diseases. Overall, we provide a comprehensive reference framework on DUB function and their roles in neuronal tissues that will be useful for future functional and evolutionary studies.

## Material and Methods

### Ethics statement

All procedures in mice were performed according to the ARVO statement for the use of animals in ophthalmic and vision research, as well as the regulations of the Animal Care facilities at the Universitat de Barcelona. The protocols and detailed procedures were evaluated and approved by the Animal Research Ethics Committee (CEEA) of the Universitat de Barcelona (our institution), and were submitted and also approved by the Generalitat de Catalunya (local Government), with the official permit numbers DAAM 6562 and 7185.

### Animal handling, tissue dissection and preparation of samples

Murine retina samples and eye slides were obtained from 2 month-old C57BL/6J (wild-type) and CD-1 (albino) animals. Animals were euthanized by cervical dislocation. Some retinas were dissected and immediately frozen in liquid nitrogen, while the rest were fixed in 4% paraformaldehyde (PFA) for 2 h at room temperature (RT), washed, cryoprotected overnight in acrylamide at 4°C, embedded in O.C.T. (Tissue-Tek, Sakura Finetech, Torrance, CA), frozen in liquid nitrogen and sectioned at -17°C.

### RNA extraction and cDNA synthesis

For each sample, retinas from three different animals were pooled. Therefore, up to 9 animals in three independent replicates were analyzed. Retinas were homogenized using a Polytron PT 1200 E homogenizer (Kinematica, AG, Lucerne, Switzerland). Total RNA was extracted using the High Pure RNA Tissue Kit (Roche Diagnostics, Indianapolis, IN) following the manufacturer’s instructions with minor modifications (increasing the DNAse I incubation step). Reverse transcription reactions were carried out using the qScript cDNA Synthesis Kit (Quanta Biosciences) following the manufacturer’s protocol.

### RT-qPCR

**Fig 1:**
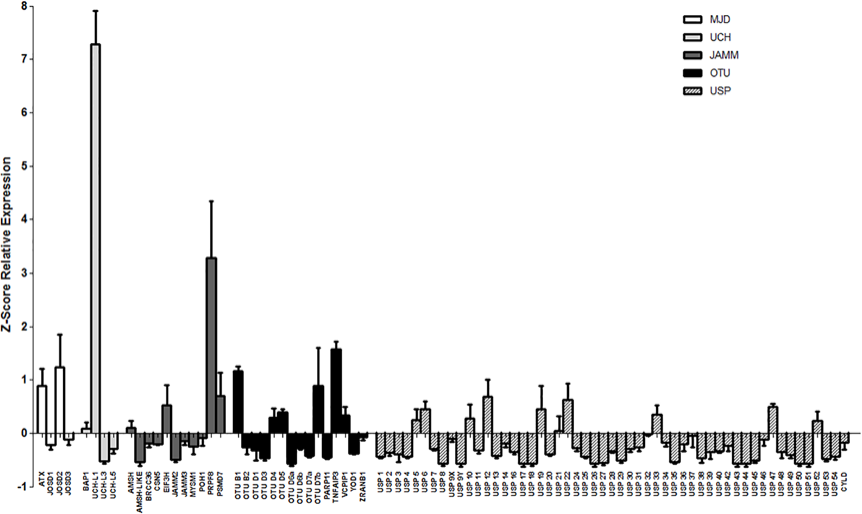
Relative expression levels of the five subfamilies of DUB enzymes. Gene expression values are the average of three independent samples (measured in three replicates), each sample contained retinas from three individuals. The expression levels are obtained as a ratio with *Gapdh* expression (used for normalization) per 10^4^. The Z-score has been calculated for the whole set of genes per each sample, and mean and standard deviation has been obtained, so that the results can be directly compared among them. Negative values indicate when genes are expressed below the global mean of the gene expression obtained in the analysis, and positive values when genes are more highly expressed. To simplify the comparison, the graph starts at the negative values, being 0 the mean value of gene expression for the whole set of genes (87 in total) in each sample. **JAMM**- JAB1/MPN/MOV34 motif proteases; **MJD**- Machado-Joseph Disease protein domain proteases; **UCH**- Ubiquitin C-Terminal Hydrolases; **OTU**- Ovarian Tumor proteases; **USP**- Ubiquitin-Specific Proteases.

Quantitative reverse transcription PCR (RT-qPCR) was performed using the LightCycler^®^ 480 SYBR Green I Master Mix (Roche Applied Science) and a LightCycler^®^ 480 Multiwell Plate 384. The final reaction volume was 10 μl. Raw data was analyzed with the LightCycler^®^ 480 software using the Advanced Relative Quantification method. *Gapdh* expression was used to normalize the levels of expression. *Rho* and *Cerkl* were considered as reference genes with high and low levels of expression, respectively, in the mouse retina. Three independent samples replicates were analyzed for each gene. Differences in gene expression levels within the same sample and between the samples were directly compared by their Z-score values. The mean and standard deviation of the Z-scores are plotted in Fig 1. The name and sequence of all the primers used for RT-qPCR and *in situ* hybridization are listed in S1 Table.

### *In situ* hybridization

For *in situ* hybridization (ISH), 16–18μm sections were recovered on commercial Superfrost Plus glass slides (Electron Microscopy Sciences, Hatfield, PA), dried 1 h at RT, rinsed three times for 10 min with phosphate-buffered saline (PBS), treated with 2 μg/ml proteinase K for 15 min at 37°C, washed twice for 5 min with PBS, and fixed with 4% PFA. Acetylation with 0.1 M triethanolamine-HCl (pH 8.0) containing first 0.25%, and then 0.5% acetic anhydride, was performed for 5 min each. Hybridization was carried out overnight at 55°C with digoxigenin-labelled riboprobes (2 μg/ml) in 50% formamide, 1 x Denhardt’s solution, 10% dextran-sulfate, 0.9 M NaCl, 100 mMTris-HCl (pH 8.0), 5 mM EDTA (pH 8.0), 10 mM NaH_2_PO_4_, and 1 mg/ml yeast tRNA. For each gene, cDNA fragments generated by RT-PCR of approximately 400-700bp were subcloned into the pGEM-T^®^ Easy Vector (Promega) and sense and antisense riboprobes were generated from the flanking T7 RNApol promoter. The name and sequence of all the primers used for RT-qPCR and *in situ* hybridization are listed in S1 Table.

After hybridization, the slides were washed in 2x SSC for 20 min at 55°C, equilibrated in NTE (0.5 M NaCl, 10 mM Tris-HCl pH 8.0, 5 mM EDTA) at 37°C, and then treated with 10 μg/ml RNase A in NTE at 37°C for 30 min. Subsequently, the sections were washed at 37°C in NTE for 15 min, twice in 2x SSC and 0.2x SSC for 15 min each, equilibrated in Buffer 1 (100 mM Tris-HCl pH 7.5, 150 mM NaCl), and blocked in Blocking Buffer (1% BSA and 0.1% Triton X-100 in buffer 1) for 1 h at RT. An anti-digoxigenin-AP conjugate antibody (1:1000; Roche Diagnostics, Indianapolis, IN) in Blocking Buffer was incubated overnight at 4°C. The sections were then washed twice in Buffer 1 for 15 min, once in Buffer 2 (100 mM Tris-HCl pH 9.5, 150 mM NaCl), and once in Buffer 2 supplemented with 50 mM MgCl_2_ (5 min each) prior to adding the BM Purple AP Substrate (Roche Diagnostics, Indianapolis, IN). For each gene, antisense and sense ISH staining reactions were processed in parallel. The reaction was stopped in 1x PBS. Sections were cover-slipped with Fluoprep (Biomérieux, France) and photographed using a Leica DFC Camera connected to a Leica DM IL optic microscope (Leica Microsystems, Germany).

### Fluorescent immunohistochemistry

For retina immunofluorescence, 16 μm sections were recovered on commercial Superfrost Plus glass slides (Electron Microscopy Sciences, Hatfield, PA), dried 30–45 min at RT, washed 10 min with PBS and blocked for 1 h with Blocking Buffer (2% Sheep Serum and 0.3% Triton X-100, in PBS 1x). Primary antibodies were incubated overnight at 4°C with Blocking Buffer. After incubation, slides were washed with PBS (3 x 10 min) and treated with DAPI (Roche Diagnostics, Indianapolis, IN) (1:300) and with secondary antibodies conjugated to either Alexa Fluor 488 or 561 (Life Technologies, Grand Island, NY) (1:300). After secondary antibody incubation slides were washed again in PBS (3 x 10min). Sections were mounted in Fluoprep and analyzed by confocal microscope (SP2, Leica Microsystems).

Primary antibodies and dilutions used were: 1:50 Rabbit anti-JOSD2 (Aviva Systems Biology); 1:50 Rabbit anti-JOSD3 (Aviva Systems Biology), 1: 50 Rabbit anti-ATXN3 (in house, a gift from Dr. S. Todi); 1:20 Rabbit anti-BAP1 (Abcam); 1:100 Rabbit anti-OTUD4 (Abcam ab106368), 1:100 Rabbit anti-PRPF8 (Abcam ab79237), 1:100 Rabbit anti-TNFAIP3 (Abcam ab74037), 1:100 Rabbit anti-UCHL3 (Abcam ab126703), 1:100 Rabbit anti-USP9X (Abcam ab19879), 1:100 Rabbit anti-USP13 (Abcam ab109264), 1:50 Rabbit anti-USP16 (Abcam ab135509), 1:100 Rabbit anti-USP22 (Abcam ab4812), 1:300 Rabbit anti-USP25 (in house), 1:250 Rabbit anti-USP28 (ABGEN AP2152b).1:500 for Mouse anti-Rhodopsin (Abcam, Cambridge, UK). Antibodies against AMSH (Biorbyt orb101007), JAB1 (Abcam ab12323), OTUB1 (Abcam ab76648), OTUD1 (Abcam ab122481), POH1 (Abcam ab8040), USP5 (Abcam ab154170) and USP45 (Novusbio H00085015) did not produce reproducible results.

### Phylogenetic analyses

Protein sequences from each enzyme group were queried in complete genome sequences of 14 animal taxa (*Homo sapiens*, *Mus musculus*, *Danio rerio*, *Petromyzon marinus*, *Branchiostoma floridae*, *Saccoglossus kowalevskii*, *Strongylocentrotus purpuratus*, *Drosophila melanogaster*, *Daphnia pulex*, *Caenorhabditis elegans*, *Lottia gigantea*, *Capitella teleta*, *Nematostella vectensis* and *Acropora digitifera*) using the HMMER 3.1 algorithm. For each analyzed enzyme family (USP, UCH, OTU, MJD and JAMM) we searched all proteins containing the Hidden Markov motifs of their catalytic region as defined in Pfam (UCH/UCH_1, Peptidase_C12, OTU/Peptidase_C65, Josephin and JAB domains, respectively). Protein domain architectures of each retrieved protein were then computed using Pfamscan 1.5 and Pfam 27 database [25] of protein domains.

We aligned the catalytic region of each enzyme family using Mafft 7 L-INS-i [26](optimized for local sequence homology), and inspected each alignment matrix manually. The most suitable evolutionary model for the analyses, selected with ProtTest 3.4 [27], was LG+ Γ. We used RaxML 8.1.1 [28] to infer Maximum Likelihood trees of each family, with 100 bootstrap replicates as statistical supports. Complete sequences, alignments and phylogenies are provided in S1–S3 Files. Manual inspection of the trees allowed us to identify subfamilies, named after their human orthologs, based on their bootstrap support and conservation of protein domain architectures.

## Results

### Expression level of deubiquitinating enzymes in the mouse retina

A RT-qPCR was performed on mouse neuroretinas to assess the expression levels of the whole set of 87 mouse genes that encode the deubiquitinating enzymes belonging to the five aforementioned families (11 JAMM, 4 MJD, 15 OTU, 4 UCH, and 53 USP genes). Two reference genes, *Rhodopsin* and *Cerkl*, were included in the analysis due to their previously reported high and low levels of expression in the mouse retina, respectively [29]. The relative expression levels have been normalized to the expression of *Gapdh*, and the Z-score was calculated for the whole set of genes per each sample, so that they could be directly compared among them and between different samples. The results (mean and standard deviation of the Z-scores per each gene) are plotted in Fig 1, ordered by DUB family. A Z-score of zero indicates the mean value of expression for all the DUBs analyzed in the retina. Thus, genes with positive values have an expression above the mean, whereas genes with negative values show less expression than the mean (e.g. most USP genes).

The results showed that *Prpf8* was the highest expressed gene from the JAMM subfamily, followed by *Eif3h* and *Psmd7*. Both *Atxn3* and *Josd2* rendered the highest expression levels within the MJD subfamily. Concerning the OTU subfamily, *Otub1* and *Tnfaip3* produced the higher expression levels, followed by *Otud7b*, *Vcpip1*, *Otud4* and *Otud5*; whereas the levels of *Otud6a* were considered as negligible. *Uchl1* was the most highly expressed gene from the UCH family (and also with respect to all DUB genes), while *Uchl3* and *Uchl5* are lowly expressed in the retina. Finally, the genes from the large USP subfamily showed the lowest level of expression among all the DUB genes. Some USPs (20%) were highly expressed and showed positive Z-scores (*Usp5*, *Usp6*, *Usp10*, *Usp12*, *Usp19*, *Usp21*, *Usp22*, *Usp33*, *Usp47* and *Usp52)* whereas 25% of the USPs showed lower levels than the mean (*Usp8*, *Usp9Y*, *Usp17*, *Usp18*, *Usp26*, *Usp27*, *Usp29*, *Usp35*, *Usp43*, *Usp44*, *Usp45*,*Usp50*, and *Usp51*) (Fig 1).

### Expression map of the DUBs in the mouse retina

Once the expression levels of all the DUB family members were assessed, we characterized and compared their expression pattern within the different layers of the mouse retina. We first decided to detect gene expression by mRNA localization using *in situ* hybridization (ISH) and then performed fluorescent immunohistochemistry of selected proteins.

**Fig 2:**
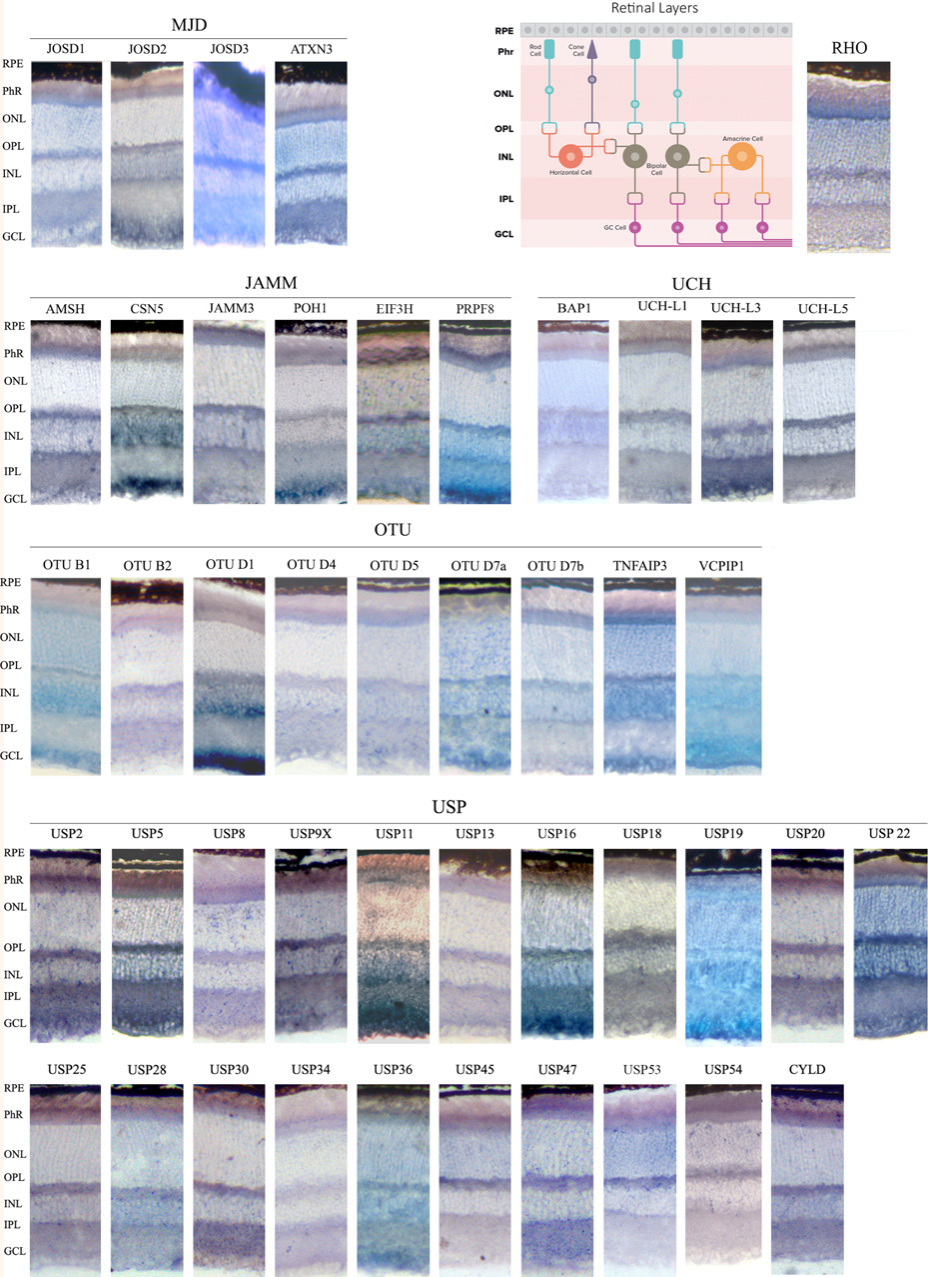
*In situ* hybridization of genes encoding DUB enzymes on retinal cryosections. Sections from wild-type C57BL/6J mouse retinas were hybridized using digoxigenin-labelled antisense riboprobes. Their corresponding sense riboprobes (negative controls) stained for the same length of time (lower panels in each row) are in the S1 Fig. The antisense *Rhodopsin* probe, which strongly labels the inner photoreceptor segment, was used as a positive control for the assay. **RPE**- Retinal pigmented epithelium; **Phr**- Photoreceptor cell layer; **ONL**- Outer nuclear layer; **OPL**. Outer plexiform layer; **INL**- Inner nuclear layer, **IPL**- Inner plexiform layer; **GCL**- Ganglion cell layer.

For ISH, antisense (AS) riboprobes against a large group of DUBs were used on mouse retinal cryosections (Fig 2). As negative controls, the corresponding sense riboprobes (S) of each gene were generated and hybridized in parallel using the same conditions (see S1 Fig). The staining time was adjusted for each set of antisense/sense riboprobes so that a maximum signal was obtained in the antisense retinal sections with minimum background in the sense counterparts (for instance, *Prpf8* and *Tnfaip3* in situs stained in much less time than *Uchl5*, *Usp8* and *Usp18*, which required half a day). *Rhodopsin* was used as a positive control because of the reported high expression in the retina and its well-known localization in the inner segment of the photoreceptors. The large USP subfamily contains 57 members in the mouse genome but only a set of genes was considered for ISH. Representative ISH results are displayed in Fig 2. Our selection criteria included genes with relevant ocular phenotypes in systematic knockdown analyses of DUBs in *Drosophila* [9] and zebrafish [30].

Most DUBs are expressed ubiquitously throughout the layers of the murine retina, which would be compatible with a general role in the neuronal cell metabolism and regulation and thus, not restricted to particular retinal neurons. Nonetheless, specific patterns of expression were detected for particular DUBs. For instance, a strong hybridization signal in the plexiform layers was observed for *Uchl3*, *Uchl5*, *Usp2*, *Usp9X*, including in some cases the inner segment of the photoreceptor layer, as detected for *Amsh*, *Josd3*, *Atxn3* and *Usp47*. Some DUBs appear to be highly expressed in the GCL (*Csn5*, *Poh1*, *Prpf8*, *Josd2*, *Otud1*, *Vcpip1*, *Usp11*, *Usp5* and *Usp19*) in contrast to the pattern generated by *Usp8*, *Usp13*, *Usp30*, *Usp45* and *Usp54*, which yielded virtually no mRNA localization signal in the ganglion cells.

Several DUB genes of the USP family (*Usp5*, *Usp13*, *Usp19* and *Usp34*) were previously reported to be differentially expressed in the Retinal Pigmented Epithelium (RPE) by transcriptome analysis [31]. To assess their specific pattern of expression, and given that pigmented cells mask positive hybridization signals, we also performed ISH on albino retinas from CD-1 mice (S2 Fig). Although these four genes are expressed in this non-neuronal layer, their expression is not restricted to the RPE. In fact, *Usp5* and *Usp19* are very highly expressed throughout the retina (Fig 2). Comparison of the retinal expression pattern for these four genes did not show any detectable difference between C57BL/6J (wild-type black) and CD-1 (albino) mice strains.

Several genes, namely *Amsh-like*, *Brcc36*, *Jamm2*, *Mysm1* and *Psmd7* (JAMM group) and *Otud3*, *Yod1*, *Zranb1* (OTU group), did not render reproducible and reliable ISHs, even though several riboprobes spanning different gene regions were used. In most cases (e.g. *Amsh-like*, *Brcc36*, *Jamm2*, *Mysm1*, and *Otud3*) we obtained very low levels of expression and the signal was too faint to be distinguished from the negative control (sense riboprobe), or the sense and antisense riboprobes both produced signals of similar intensity. The ISH results of these genes are not included here.

Taking the ISH results together, we drew an atlas of expression for DUBs in the retina of adult mouse. In general, all analyzed genes except *Otud1* are expressed in the photoreceptors, and their mRNAs are localized in a wide range of intensities in the inner segment (perinuclearly) and the outer plexiform layer. Among layers, the GCL showed the most different pattern of gene expression. Notably, some DUBs, such as *Usp45*, *Usp53* and *Usp54*, are only detected in photoreceptors (PhR -inner segments, ONL (photoreceptor nuclei and perinuclei) and OPL (photoreceptor synapsis), whereas nearly no hybridization could be detected in the rest of retinal layers, which would suggest specific roles for these DUBs in this highly specialized photosensitive cells.

**Fig 3:**
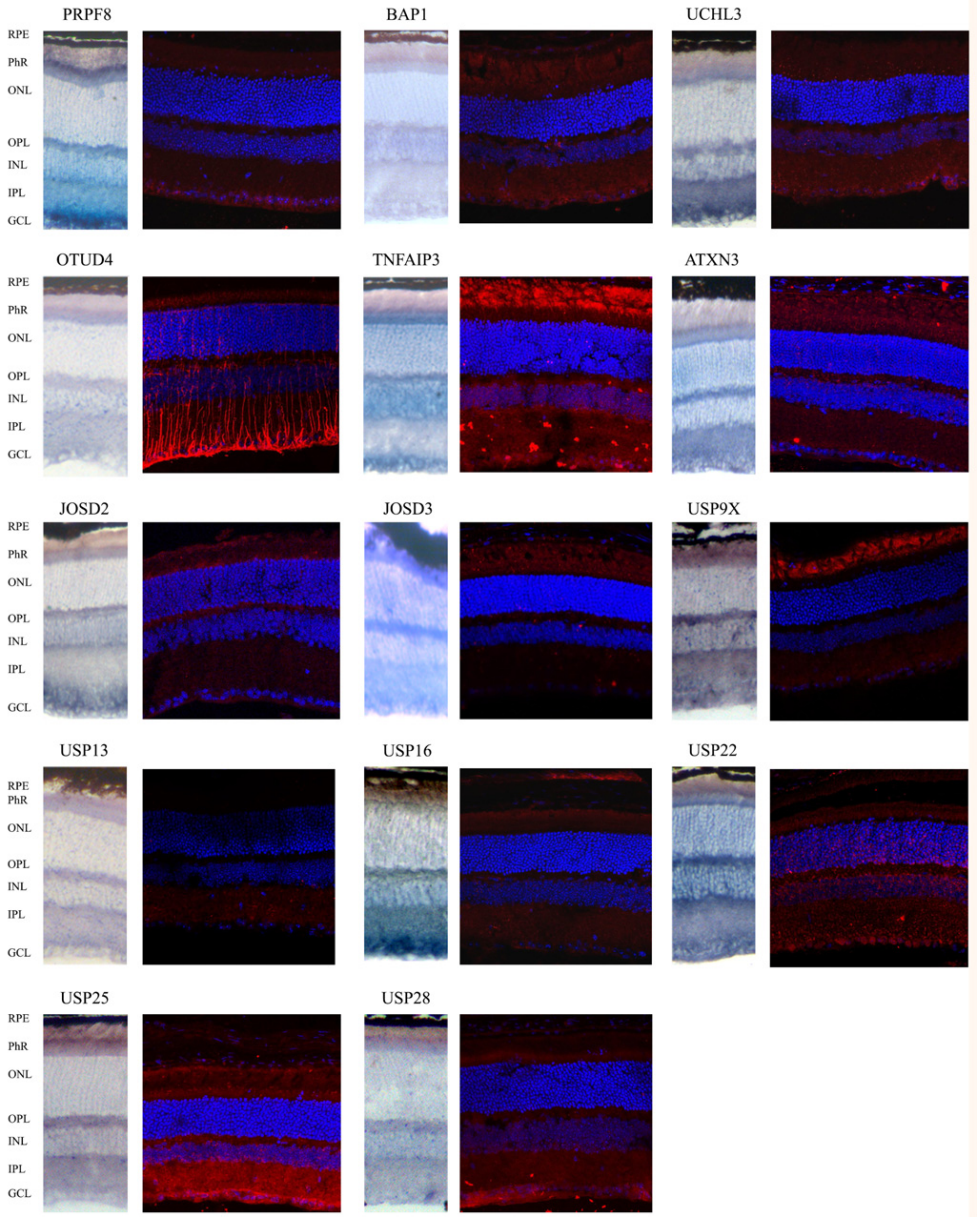
Comparison of mRNA and protein immunodetection of selected DUBs in mouse retinal cryosections. Most analyzed genes render a consistent expression pattern when comparing mRNA and protein localization in the wild type mouse retina. The merge immunohistochemistry show DUBs immunodetected in red, and nuclei counter-staining with DAPI (in blue). Details in S3 Fig. **RPE**- Retinal pigmented epithelium; **Phr**- Photoreceptor cell layer; **ONL**- Outer nuclear layer; **OPL**. Outer plexiform layer; **INL**- Inner nuclear layer, **IPL**- Inner plexiform layer; **GCL**- Ganglion cell layer.

These ISH results prompted us to confirm and define more accurately protein localization within the retinal cell layers by fluorescent immunohistochemistry, since in cells with a highly specialized morphology, mRNA and protein localization might be different (e.g. the mRNA of rhodopsin is localized in the ribosome-rich photoreceptor inner segment whereas the protein is highly abundant in the membranous disks of the outer segment). We selected a group of DUBs for immunohistochemistry based on: i) particular ISH patterns, ii) relevance for eye phenotype in animal models, iii) putative functional diversification in phylogenetically closely related enzymes (see next section), and iv) antibody commercial availability and affinity. We selected 21 DUBs (the list of genes is detailed in the Material and Methods), of which 14 immunodetections rendered a reproducible and reliable signal (Fig 3 and S3 Fig).

Overall, the immunodetection confirms the ISH results since protein is detected in the same retinal cells than mRNA (Fig 3). Comparing RT-qPCR to ISH and immunohistochemistry results, high levels of retinal expression correlated with a ubiquitous expression pattern. Besides, some protein locations are worth mentioning as indicative of distinct functions in specific cellular compartments. For instance, OTUD4 is strongly detected in the axonal processes of bipolar and other retinal cells, supporting its involvement in neurodegeneration in human [32]. USP25 is mainly detected in the inner plexiform and ganglion cell layer; while USP9X and TNFAIP3 are particularly detected (but not exclusively) at the outer photoreceptor segment. Besides, USP22 is localized in the nucleus of ganglion cells, and perinuclearly in the rest of retinal neurons. For details, merge and separate immunodetection images, see S3 Fig.

### DUB phylogenetic analysis, protein domain architecture and neuronal phenotypes

To provide a rational framework for gene expression patterns in extended families, it is crucial to have an understanding of the origin and phylogenetic closeness between the different DUB genes. Therefore, we performed a bioinformatic survey of DUB protein sequences across animal taxa. A recent phylogenetic analysis of the ubiquitin system across eukaryotes already showed that a massive expansion of ubiquitin ligases and proteases, which involves innovation and incorporation of new protein domains, occurred at the origin of animal multicellularity [24]. This was likely associated with the diversity of proteins and protein roles in different cell types. We here provide a comprehensive picture of the DUB families during the diversification of metazoans, related to previously described neuronal function, with an emphasis on eye and retinal phenotype.

**Fig 4:**
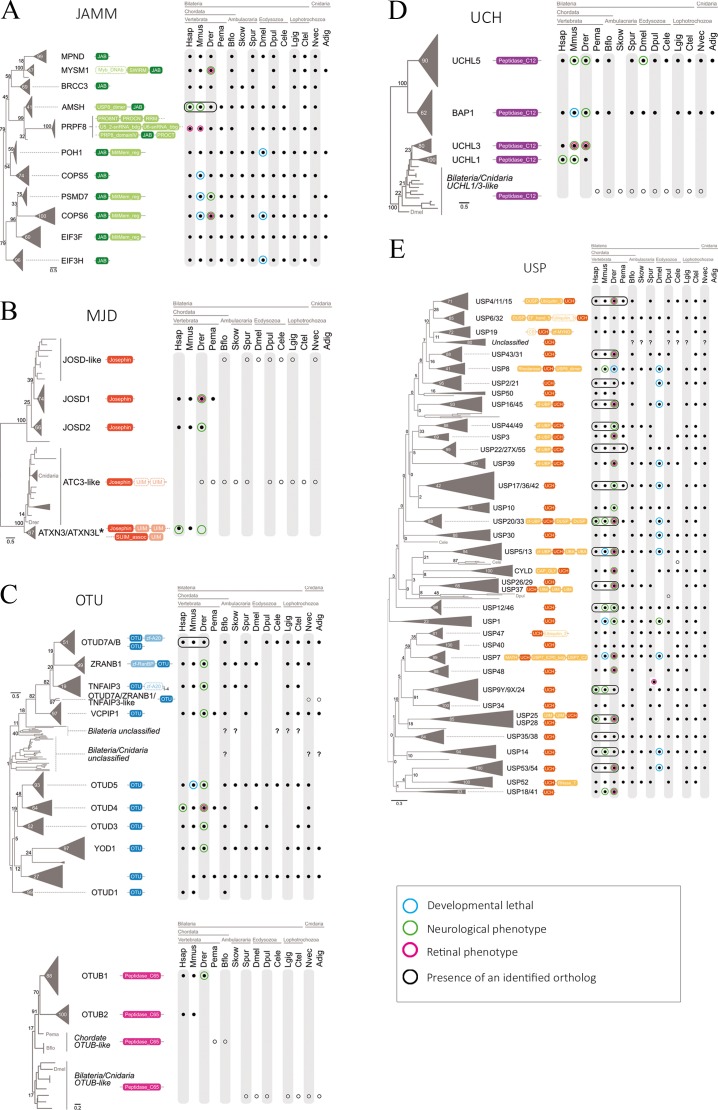
Phylogenetic analysis of DUB genes and neuronal/retinal phenotype. Protein sequences from the catalytic region of each enzyme group were queried in complete genome sequences of 14 animal taxa and aligned. The protein domain architectures including the catalytic and accessory domain motifs are represented next to each DUB member (A, JAMM; B, MJD; C, OTU; D, UCH; and D, USP). Black dots indicate presence of the ortholog, whereas white dots indicate homologs that cannot be confidently assigned to a DUB type (see Results). Question marks represent statistically supported clades of uncharacterized DUBs. DUB sequences that are highly similar and cluster closely together appear collapsed under a common name. In general, invertebrates have a single representative member of the collapsed branch, whereas vertebrate genomes show one member of each paralog (species circled in black). *Acropora digitifera* USP homologs were excluded from the analysis as they impaired the resolution of the USP phylogeny. Genes reported to produce an abnormal neuronal phenotype when mutated are circled in magenta, whilst genes producing abnormal eye or retinal phenotype are circled in green. Genes whose mutation is lethal during developmental stages are circled in blue. An schematic summary of the DUB mRNA localization in the mouse retina (from ISH) is also presented next to the corresponding family. The intensity of the color indicates hybridization signal intensity. Retinal layers appear indicated as in Fig 2.

Completely sequenced genomes from 14 species (from cnidarians to vertebrates) were queried with the catalytic region of each enzyme family (as defined in Pfam) in search of orthologs. Phylogenetic trees were generated using the retrieved sequences, and the statistical support for each node is also indicated (Fig 4A, 4B, 4C, 4D and 4E). For the sake of clarity, protein nomenclature is according to human DUBs. Highly similar sequences that expanded recently (during the pre-vertebrate/vertebrate expansion) and clustered together appear collapsed. The presence of an identified ortholog in each species/clade is represented with a black dot. Vertebrate species that present all the paralogs in a collapsed branch are circled in black. White dots mark the presence of homologs that could not be confidently assigned to a characterized DUB type, either because they are sister-group to various known DUB paralogs (and therefore represent the pre-duplication homolog), or because statistical support is too low to confidently cluster them with a specific ortholog. Question marks represent statistically supported clades that cannot be assigned to any known DUB (or group of paralogous DUBs). Protein motifs (as defined in Pfam) including the catalytic domain are drawn next to each branch to illustrate the diversity/conservation in protein architecture within each family. For detailed and complete phylogenetic trees, see S3 File.

Notably, the phylogenetic distribution of OTU DUBs reveals two different groups that appeared at the origin of eukaryotes OTUs with peptidase C65 domains (OTUB1 and OTUB2 in animals) and those with OTU domain [24] (Fig 4D). Given that i) these two catalytic domains diverged long before the origin of metazoans, ii) OTUB1/B2 protein domain architectures are clearly different from the other OTUs, iii) OTUB homologs are present in all metazoan clades, and iv) this split does not occur in any other family of DUBs, a new classification might be in order to acknowledge a new subfamily of DUBs.

The JAMM family has clear sequence assignment in all the analyzed animals, even though some species have secondarily lost some DUB members, e.g. *Acropora* (cnidarian), *C*. *elegans* (nematode), *Drosophila* (insect) *Saccoglossus* (hemichordate), and *Petromyzon* (sea lamprey, an early-branching vertebrate). These species also show specific gene loss for other DUB families, pointing to a divergent evolution in their lineages.

**Fig 5:**
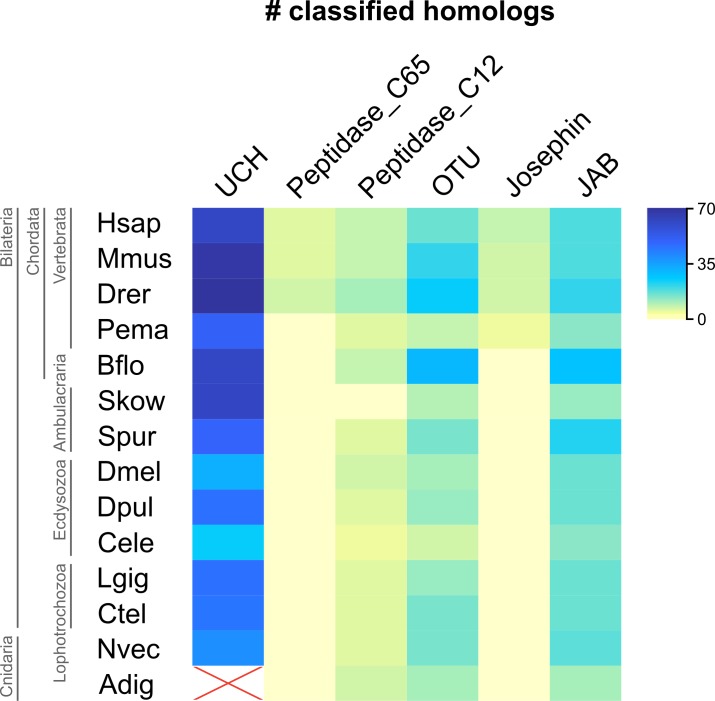
Counts of classified DUB homologs. Heatmap representing the number of classified genes in each analyzed genome. Increasing intensity reflects increasing number of genes. Only orthologs marked with black dots in Fig 4 are considered. *Acropora digitifera* USP homologs, excluded from the phylogenetic classification, are marked as not analyzed (NA).

On the other hand, a clear expansion within each DUB family has occurred in the vertebrate lineage (Figs 4 and 5). When these duplicated members have rapidly diverged, the DUB protein sequences are in separate branches, but the common ancestry becomes evident since a single ancestral ortholog is present in the rest of clades (white dots in Fig 4). This is the case within the UCH (UCHL1 and UCHL3) and MJD families (JOSD1 and JOSD2). When the duplicated sequences have diverged but still branch closely together in the phylogenetic tree, the vertebrate paralogs have been collapsed into a single branch (black circles in Fig 4). This is particularly evident for USPs, where we can identify a single ancestral sequence in all invertebrate clades whereas several members are present in vertebrates (e.g. USP4/11/15…). Note that in the case of USP 18/41, a duplication event occurred only in the case of humans; as it is a single-species case, we have not included any black box on the figure. The *ATXN3* gene deserves specific mention, since its close paralog, *ATXN3L*, is a retrogene, that is, a gene generated by a very late retrotransposition event within the primate lineage.

The DUB gene expansion in animal phylogeny is visually summarized in the heat map of Fig 5. Color intensity reflects the number of genes per genome. It becomes evident that a burst of gene expansion within all DUB families was at the basis of the vertebrate lineage. Nonetheless, the innovation in the protein architectures with the acquisition of new domains accompanying the DUB catalytic signatures, pre-dates the origin of vertebrates in all the analyzed families, as vertebrate-like domain arrangements are often identified in other animal clades.

To complement our DUB expression study in the retina and in order to suggest relevant genes for hereditary visual disorders, we have compared the reported DUB mutant phenotypes of several animal models and human diseases, and viewed them under our new phylogenetic framework. We have specifically searched for early developmental lethality, neuronal phenotype and retinal alterations when available (Fig 4A, 4B, 4C, 4D and 4E). In the cases of neuronal phenotype, there is an accompanying alteration in the eye. However, most phenotypic assessment in the eye report only gross alterations, but a detailed retinal study has not yet been described for most animal models. For a detailed phenotypic trait list, see S2 Table and references therein.

In general, we observe that families with ancestral genes that have not been expanded in vertebrates (particularly the JAMMs) have a ubiquitous expression profile in the retina, suggesting a basic cell function. Moreover, mutations of these real orthologs produce consistent phenotypes through the analyzed taxa, arguing in favor of functional and evolutionary conservation. In contrast, for close paralog DUB genes arisen by duplication events in the vertebrate lineage, different patterns of retinal gene expression are often observed. A good example is OTUD7A/B (with one ancestral gene in most animals, and expanded in vertebrates), where OTUD7A is more highly expressed in the GCL and plexiform layers, whereas OTUD7B is more expressed in the photoreceptors. Similarly, UCHL3 and UCHL1 (both specific to vertebrates and associated to neuronal phenotypes) are expressed differently. Notably, UCHL3 (detected in the GCL and photoreceptors by ISH and immunodetection) produces eye specific retinal alterations, supporting subfunctionalization of these two paralogs. Other examples are included in the discussion.

## Discussion

The ubiquitin-proteasome system (UPS) is currently viewed as one of the most dynamic and versatile cell regulators in eukaryotes. Perturbations of this system are known to be at the basis of many human disorders, particularly cancer and neurodegeneration [5,33]. Due to their ability to deconjugate ubiquitin, DUBs play a major regulatory role in the UPS. The disruption of DUB genes has dramatic consequences for the animal taxa analyzed, either during development or in adult stages, as shown by reports of the systematic DUB knockdown in zebrafish embryos and flies [9,30].

In mammals, several comprehensive surveys of DUBs have been reported resulting in: *in silico* inventories of the DUBs in the human genome [22,34]; identification of protein interactors by cell-based proteomics analysis [8]; studies of subcellular localization [1]; functional involvement in maintaining genome integrity in cells [35]. A recent review reported the expression levels of DUBs in human organs and the disease phenotypes associated to DUB mutations in humans and animal models [23]. Despite their importance, detailed expression and functional analysis for most DUBs on particular tissues or organs, such as the retina, is still missing. We here aimed to fill this gap and produced a descriptive landscape of the expression of the complete set of DUBs in the mouse retina by combining mRNA and protein localization. We have also delineated a detailed evolutionary history of the different DUB families using phylogenetic analysis. We compared their protein domain architectures, and considered the neuronal and retinal phenotypes associated to each gene mutation/knockdown. We thus provide a reference framework for researchers interested in this visual tissue, either in physiological or in disease conditions, and suggest new avenues of research in DUBs as excellent candidates for retinal/visual hereditary disorders.

### Differential levels of DUB gene expression in the adult mouse retina

Some genes that are barely expressed in the mouse retina (e.g. *Brcc36*, *Poh1*, *Bap1*, *Otub2*, and *Usp44*) are reported to be induced in replicative cells instead, being recruited to DNA damage sites where they regulate DNA repair and mitosis checkpoints [35]. These results are consistent with the fact that the adult retina is mostly formed by differentiated cells.

Among the genes highly expressed in the adult retina, *Uchl1*, *Atxn3*, *Otub1*, *Usp6*, *Usp22* and *Usp33* are also highly expressed in the brain [23]. In fact, *Uchl1*, *Otub1* and *Atxn3* are involved in neurodegenerative diseases in human, namely Parkinson's disease and cerebellar ataxia [6,36], thus indicating a relevant role in neurodegeneration. Our ISH results showed ubiquitous mRNA localization through all the retinal layers for these three genes, supporting a possible basal function in the retina. On the other hand, other DUB genes that are highly expressed in the brain [23], such as *Mysm1*, *Usp26*, *Usp29*, *Usp35* and *Usp51*, were barely expressed in the adult mouse retina; and genes that showed very low levels of expression when analyzed by qPCR within this work such as *Usp2*, *Usp25*, *Usp45*, *Usp53* and *Usp54* rendered eye phenotype when knocked-down in zebrafish [30]. Note that we performed RT-qPCR in whole adult neuroretinas at P60, and the role of these genes during development might be more relevant than in the adult stage. It is also worth noting that *Usp45*, *Usp53* and *Usp54* did show layer specificity, as they were mainly expressed in the photoreceptors (PhR inner segment, ONL and OPL), suggesting a specific role for these genes in photoreceptors and underscoring their role as potential candidates for visual disorders.

Immunohistochemical localizations also point to specific functions for some DUBs, e.g. OTUD4 is highly localized in axons; TNFAIP3 is highly expressed in the photoreceptor outer segment and GCL, and USP22 protein localization is mainly nuclear and perinuclear, thus suggesting that these genes may be good candidates for particular retinal phenotypes.

### Phenotypic comparison of DUB mutants and gene expression profiles under the new evolutionary framework

Animal models have been generated by gene disruption (mouse) or knockdown (*Drosophila*, zebrafish) for some DUBs. When the DUB function is extremely relevant for cell cycle or cell differentiation, a lethal/early and extensive neuronal phenotype is consistently apparent in different organisms, as it is the case for most JAMMs and several USPs (see Fig 4 and S2 Table). In vertebrates, when some mutants show neuronal/brain affectation, a retinal/eye phenotype is also one of the accompanying phenotypic traits (examples are found in all the families). In fact, multiple vertebrate USP genes are present in paralogs (probably arising from the several rounds of genome duplication at the base of their linage), whereas their invertebrate relatives have a single homolog (black boxes in Fig 4). Therefore, it is not surprising that most USP knockdowns are lethal in *Drosophila* (where only a single member is present), whereas in vertebrates, the mutant phenotype mostly affect specific tissues, probably related to the larger panoply of USP members and a higher functional diversification. For instance, in zebrafish the knockdown of *Usp33* (whose close relative homolog is *Usp20*) alters the nervous system development including the eye [9], which is consistent with a reported subcellular localization associated to microtubules and centrosomes; whereas the knockdown of the only member USP20/33 in *Drosophila* is lethal. Something very similar occurs with the knockdown of *Usp53* (whose close relative homolog is *Usp54*), which affects brain and eye development in zebrafish, whereas the knockdown of the single USP53/54 member is lethal in *Drosophila* (Fig 4B and S2 Table). For all the DUB families, orthologs share both high sequence similarities and consistent mutant phenotypes in vertebrates; overall, pointing to their functional conservation and supporting mouse and zebrafish models for assessing DUB roles in the human retina.

The knockdown phenotypes in different species are sometimes partially overlapping between neuronal and retinal alterations, probably due to subfunctionalization of different paralogs due to duplication events. For instance, *Usp5* and *Usp13* (encoding enzymes that expanded and diverged in the vertebrate lineage, and sharing 59.5% amino acid identities in human) showed a distinct pattern of expression in the mouse retina, with *Usp5* being highly expressed in the GCL in contrast to *Usp13*, which is barely expressed in this layer and the protein is mostly localized in the inner plexiform layer, thus indicating different roles despite sequence similarity. The knockdown of any of them severely alters zebrafish embryonic development and causes neurodegeneration (even though only the *Usp5* knockdown showed a clear eye phenotype), whereas in *Drosophila* the disruption of the single member *Usp5/13* alters eye development by increasing photoreceptor apoptosis, thus recapitulating neurodegeneration and retinal phenotype. Similarly, the close paralogs *Usp16* and *Usp45* have a contrasting expression pattern, with the former in GCL and plexiform layers, and the latter restricted to the photoreceptor cell layer, supporting again subfunctionalization or neofunctionalization of the vertebrate paralogs. Of note, the knockdown of Usp45 in zebrafish shows reduced eyes. Interestingly, *fat facets* (the ortholog of *Usp9X*, involved in endocytosis in the Notch pathway) limits the number of photoreceptors in *Drosophila* [37], while the human homolog *USP9X* has been involved in neurodegeneration, mental retardation, epilepsy and autism, as well as in cancer [38], but not yet in visual disorders. Nonetheless, the strong immunodetection in the outer segment of photoreceptors would indicate that it is also a good candidate for retinal dystrophies.

Finally, the only DUB-related gene that has been directly involved in human inherited retinal degeneration and causative of autosomal dominant Retinitis Pigmentosa is *PRPF8*, the JAMM-family member with the highest level of expression in the retina. Notably, PRPF8 (which is not properly a DUB since it is catalytically inactive) forms part of the splicing machinery [39]. Even though *PRPF8* is a housekeeping gene, its haploinsufficiency might cause a shift in the splicing patterns, which in turn alters the highly sensitive photoreceptors and triggers their apoptosis. Knock-in mice bearing human missense mutations also display retinal degeneration, thus strengthening the significance of this JAMM-gene in the retina [40].

## Conclusions

In summary, our results show that data on the expression of the deubiquitinating enzyme gene family cannot be directly extrapolated between tissues or organs since cell requirements might be completely different, particularly in highly specialized and structured tissues, such as the retina. Therefore, in large families of seemingly redundant enzymes (such as DUBs) the integration of systematic expression maps together with a robust phylogenetic analysis and available phenotypic information provides an insightful reference framework for further functional characterization. This framework may be helpful for researchers working in the ubiquitin-related field as well as for those working in the molecular bases of neurological and retinal disorders.

## Supporting Information

S1 Fig *In situ* hybridization of genes encoding DUB enzymes on mouse retina cryosections, with the comparison between antisense and sense riboprobes. (PDF)

S2 Fig *In situ* hybridization of genes encoding DUB enzymes on CD-1 (albino) mouse retina cryosections. (PDF)

S3 Fig Fluorescent immunohistochemistry of selected DUBs. (PDF)

S1 File Zip file containing the DUB catalytic domain sequences (per family) used for the phylogenetic analysis in FASTA format. (ZIP)

S2 File Zip file containing the sequence alignments obtained per each DUB family. (ZIP)

S3 File Zip file containing the complete phylogenetic trees with their corresponding bootstraps. (ZIP)

S1 Table Sequences of the primer pairs used in the reverse transcriptase Real Time qPCR and *in situ* hybridization. (PDF)

S2 Table Mutant neuronal and retinal phenotypes in different animal models and human listed per DUB family and gene. (PDF)
