# Supplementary figures and images for: Expression Atlas of the Deubiquitinating Enzymes in the Adult Mouse Retina, Their Evolutionary Diversification and Phenotypic Roles
Source: PLoS One. 2016 Mar 2;11(3):e0150364. doi: 10.1371/journal.pone.0150364 (PMC4774998; doi:10.1371/journal.pone.0150364)

JAMM

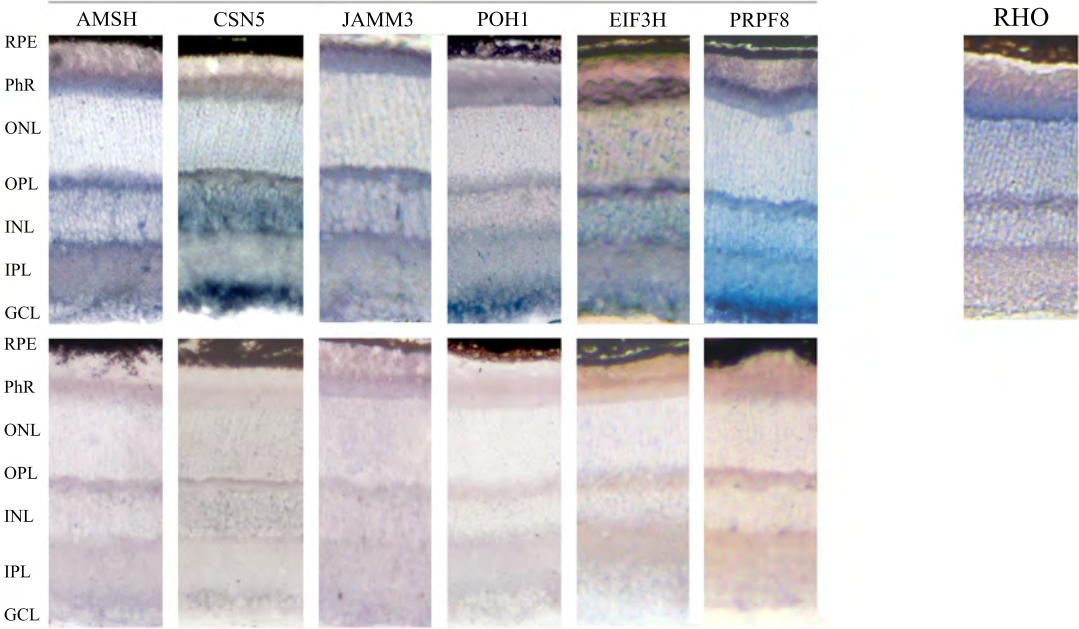

MJD

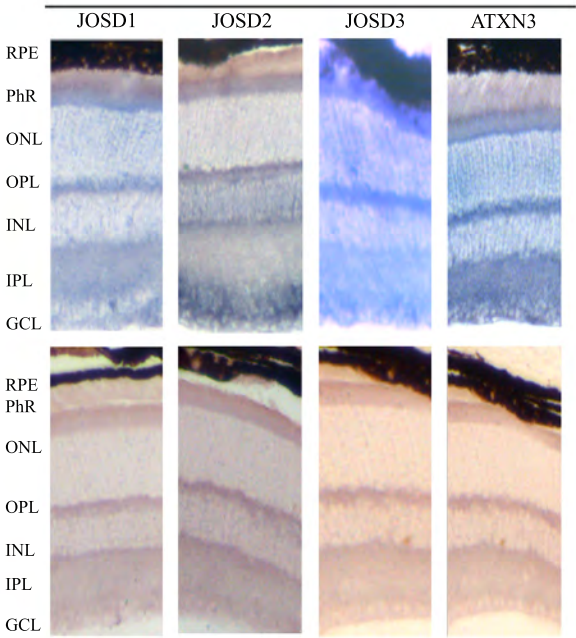

UCH

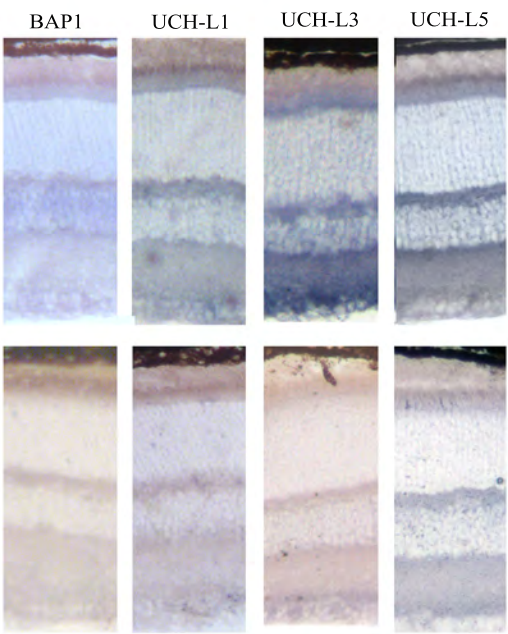

OTU

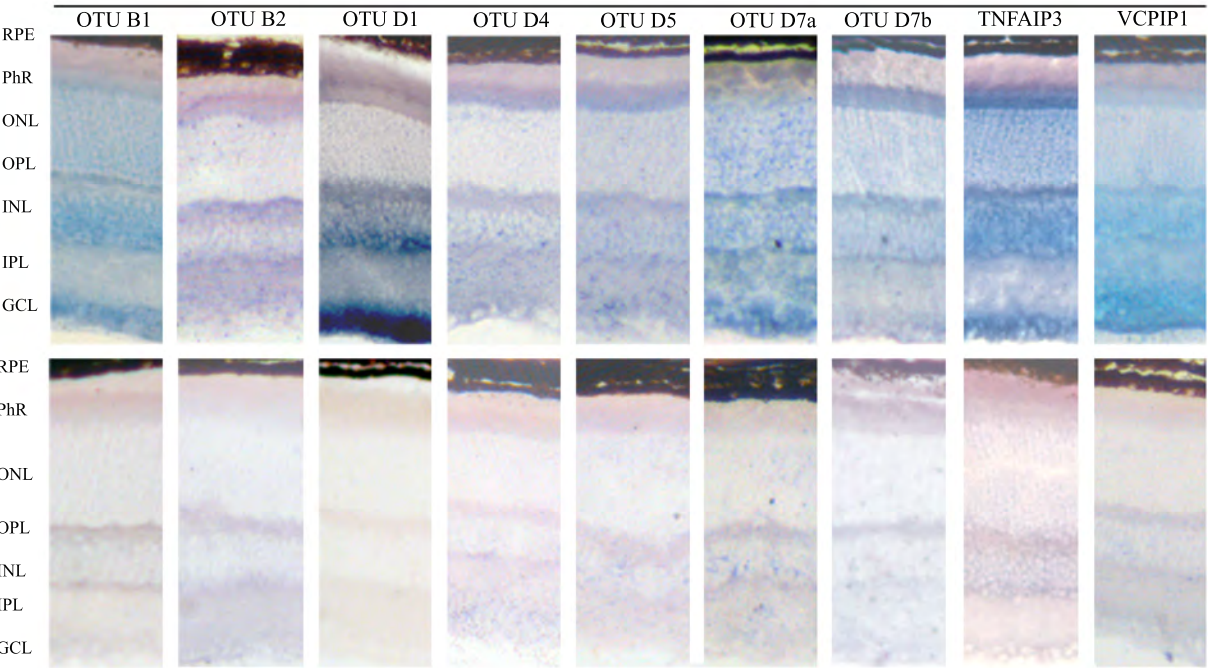

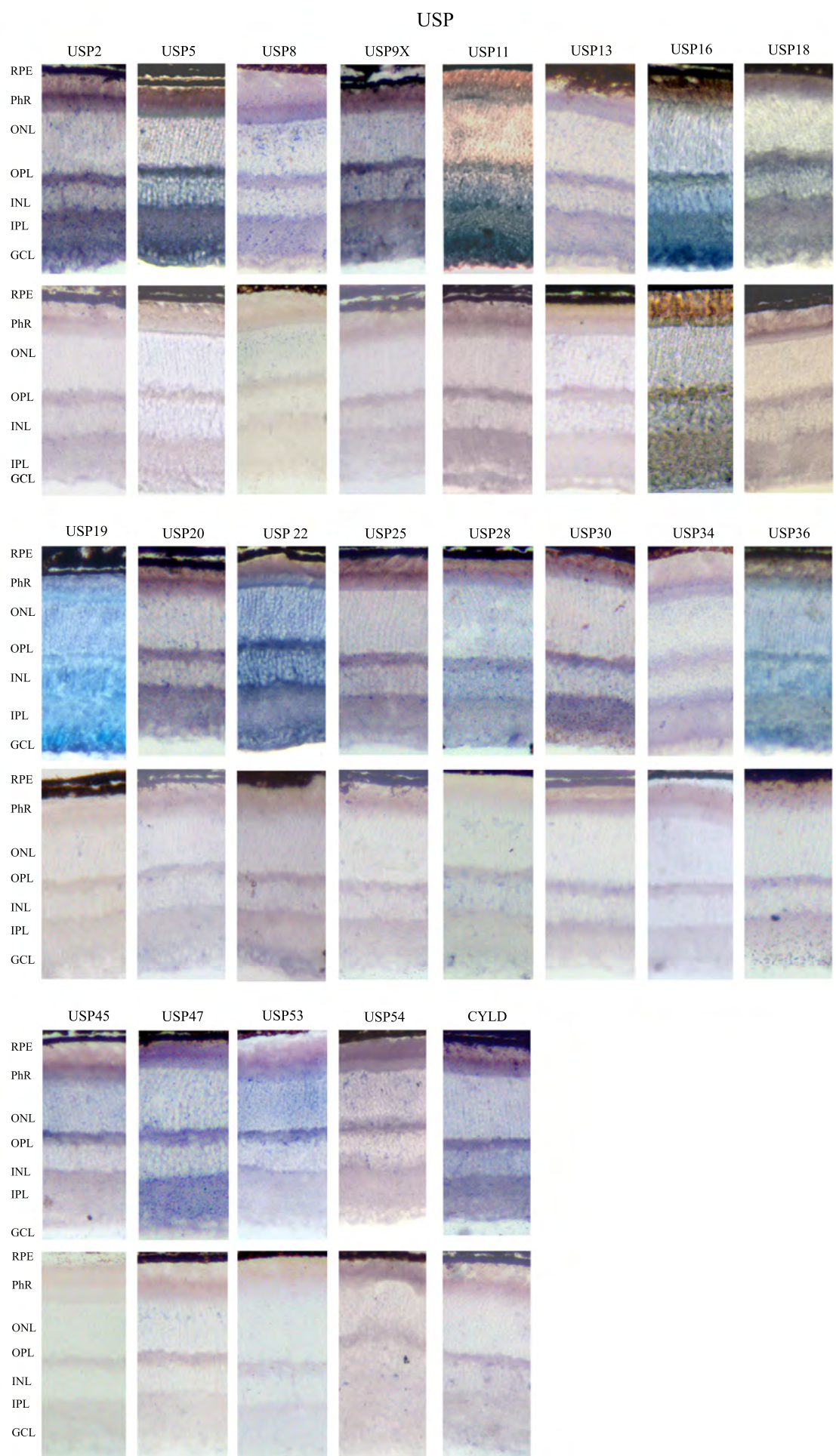

Supplement: S1 Fig — (PDF) [file pone.0150364.s001.pdf]

Peptidase\_C65 RAxML

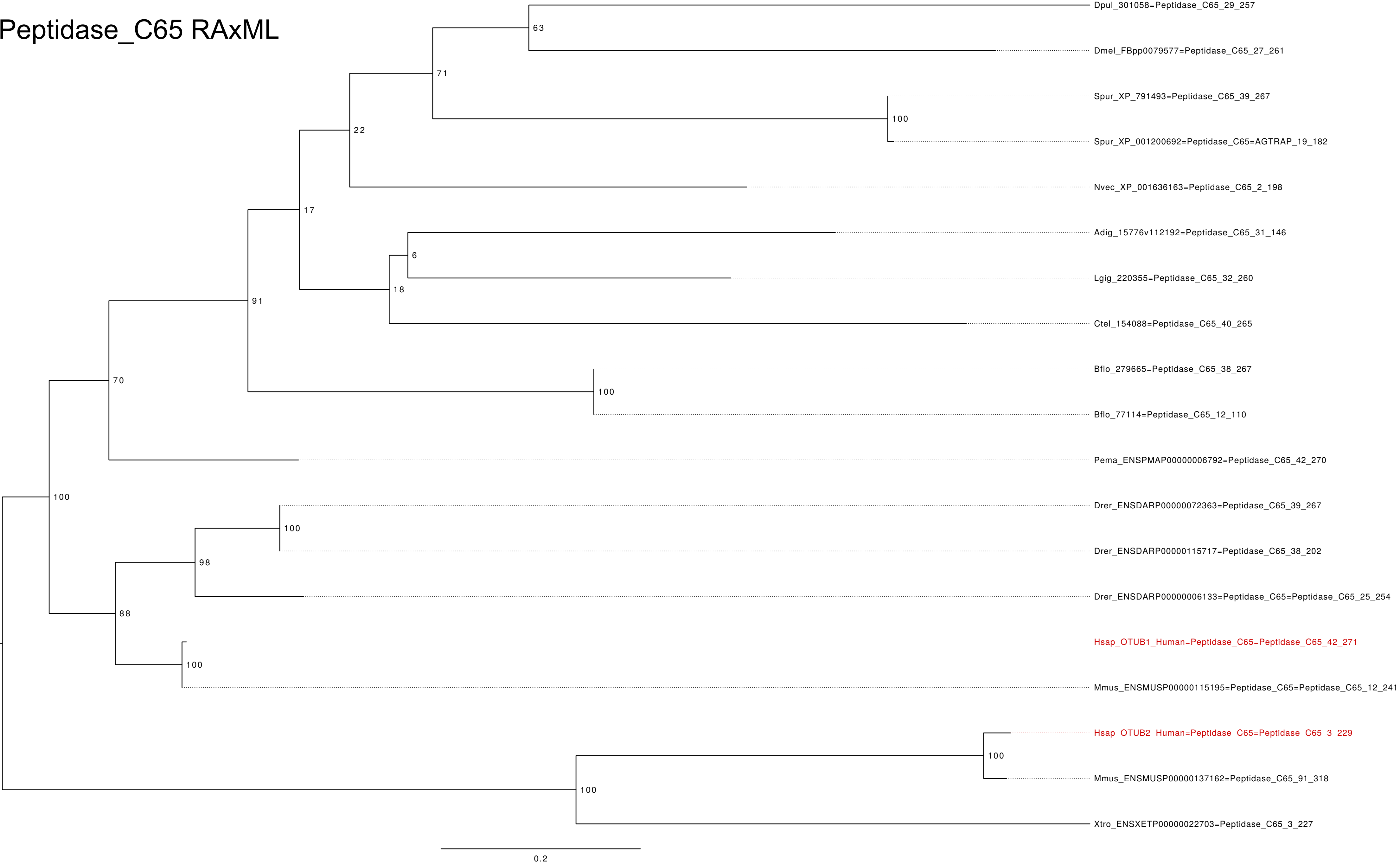

Supplement: S3 File — (ZIP) [file pone.0150364.s006.zip › sm_trees/p65.04.bsbt_Arqs.tre.pdf]

# Josephin RAxML

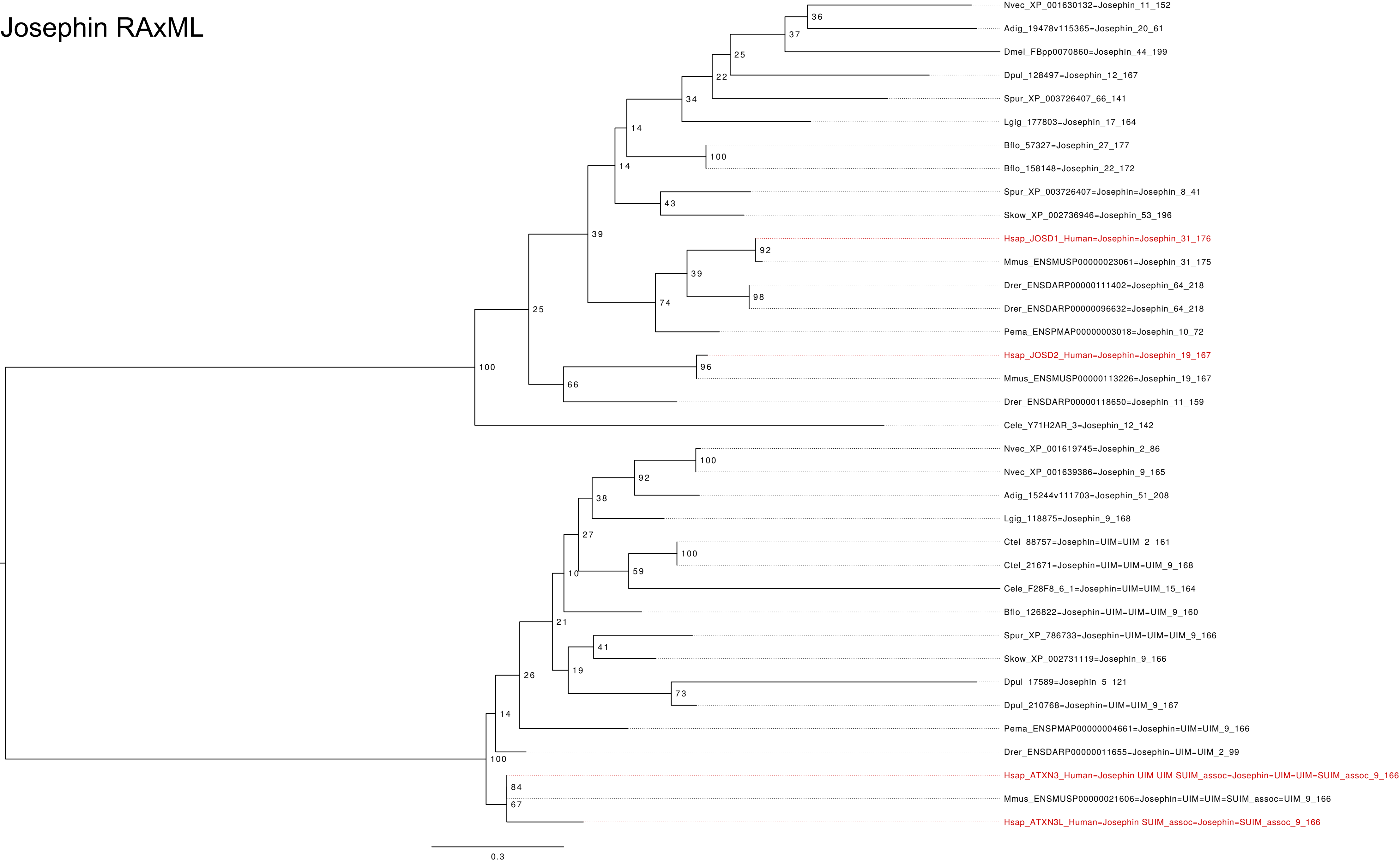

Supplement: S3 File — (ZIP) [file pone.0150364.s006.zip › sm_trees/jos.04.4nox.bsbt_Arqs.tre.pdf]

Peptidase\_C12 RAxML

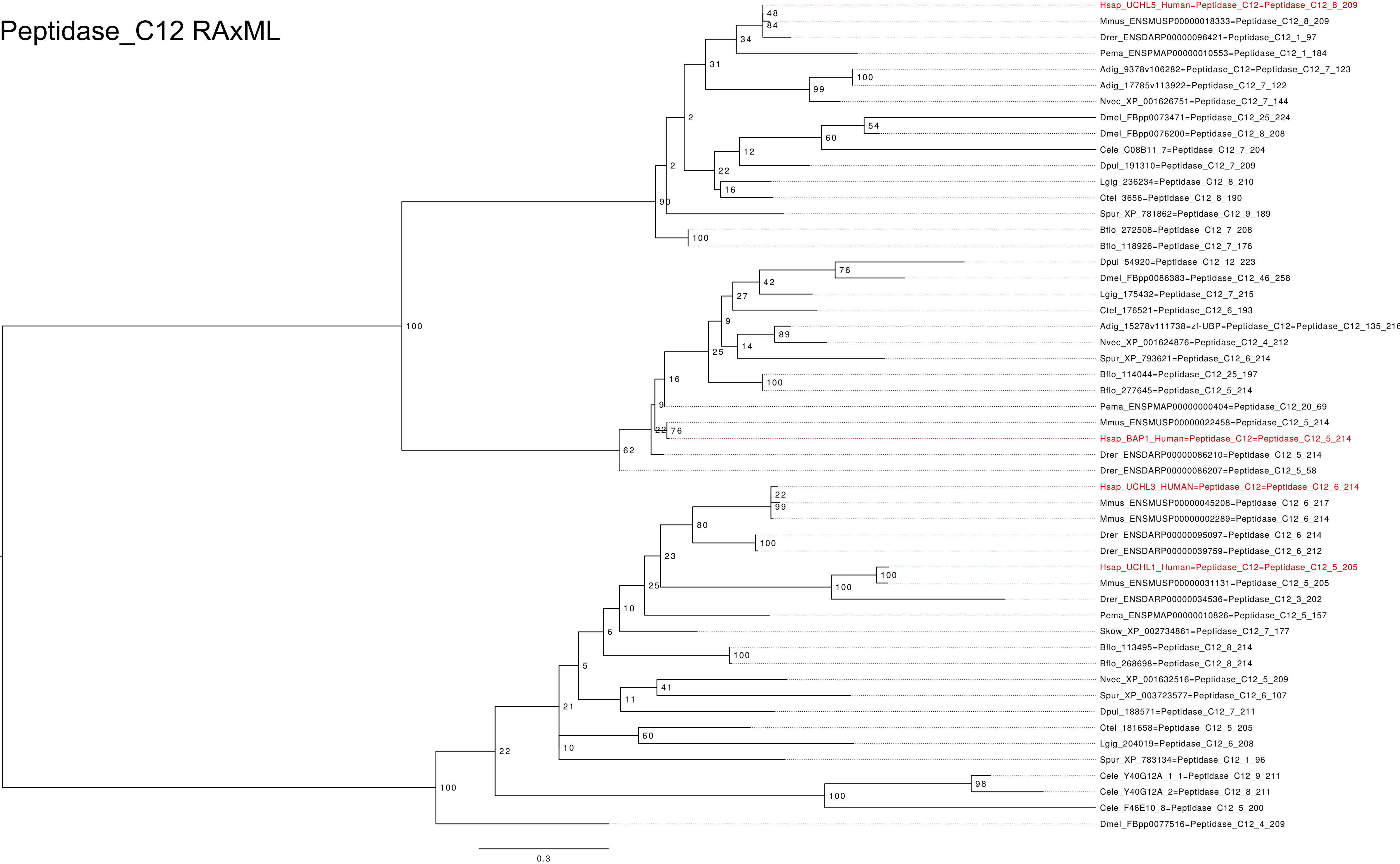

Supplement: S3 File — (ZIP) [file pone.0150364.s006.zip › sm_trees/p12.04.bsbt_Arqs.tre.pdf]

# OTU RAxML

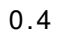

Supplement: S3 File — (ZIP) [file pone.0150364.s006.zip › sm_trees/otu.04.bsbt_Arqs.tre.pdf]

JAB RAxML

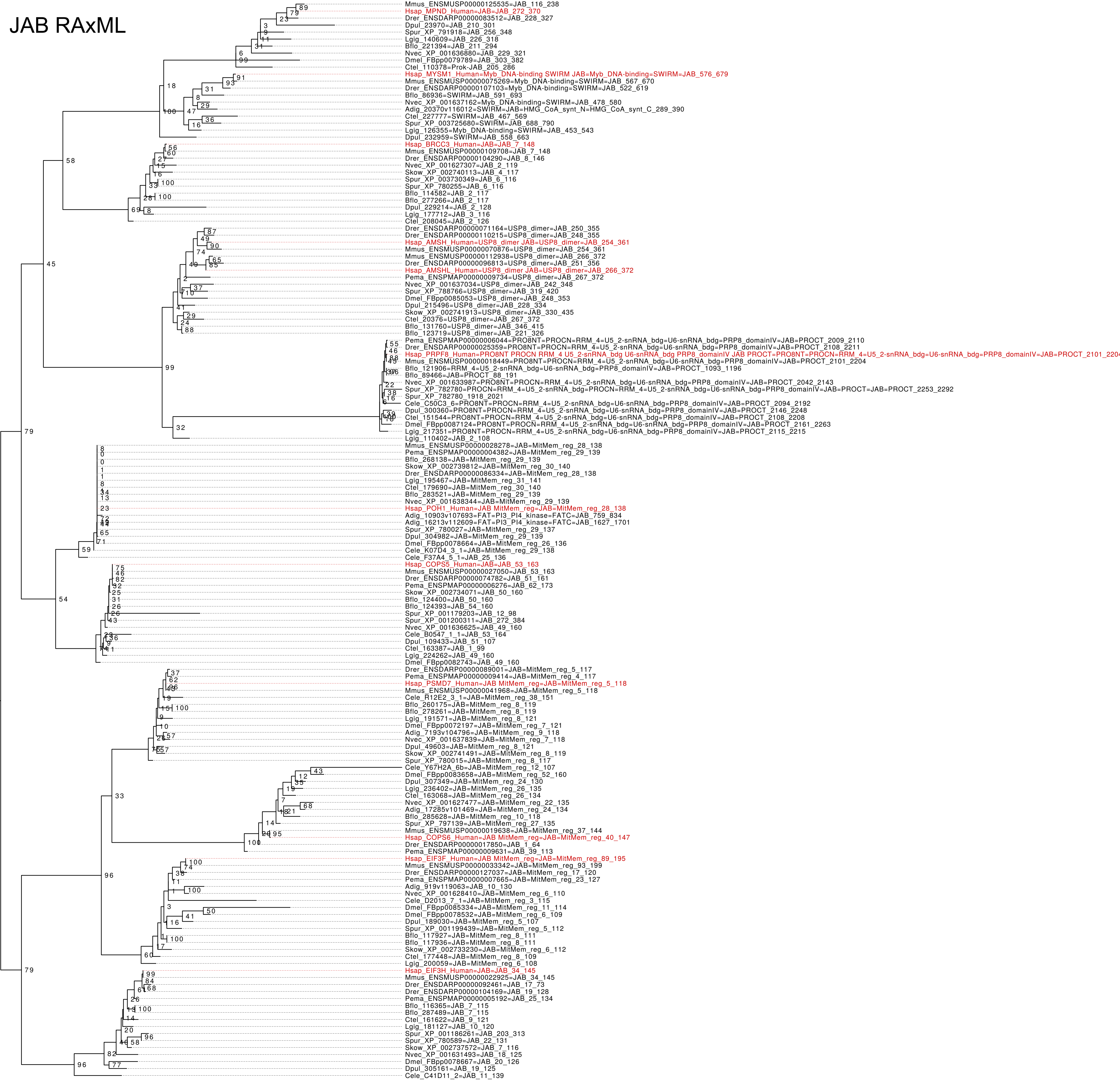

0.5

Supplement: S3 File — (ZIP) [file pone.0150364.s006.zip › sm_trees/jab.04.bsbt_Arqs.tre.pdf]
