# Supplementary material for: Expression Atlas of the Deubiquitinating Enzymes in the Adult Mouse Retina, Their Evolutionary Diversification and Phenotypic Roles
Source: PLoS One. 2016 Mar 2;11(3):e0150364. doi: 10.1371/journal.pone.0150364 (PMC4774998; doi:10.1371/journal.pone.0150364)
Supplement: S2 Fig — (PDF) [file pone.0150364.s002.pdf]

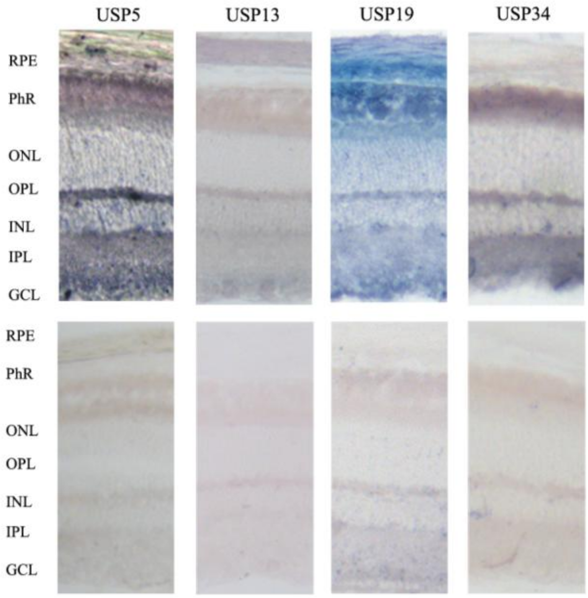

Supplementary Figure 2. *In situ* hybridization of genes encoding DUB enzymes on CD-1 (albino) mouse retina cryosections, using digoxigenin-labelled antisense riboprobes (top panels in each row) and their corresponding sense riboprobes (negative controls) stained for the same length of time (lower panels in each row). **RPE-** Retinal pigmented epithelium; **Phr-** Photoreceptor cell layer; **ONL-** Outer nuclear layer; **OPL**. Outer plexiform layer; **INL-** Inner nuclear layer, **IPL-** Inner plexiform layer; **GCL-** Ganglion cell layer.
