# Supplementary material for: Expression Atlas of the Deubiquitinating Enzymes in the Adult Mouse Retina, Their Evolutionary Diversification and Phenotypic Roles
Source: PLoS One. 2016 Mar 2;11(3):e0150364. doi: 10.1371/journal.pone.0150364 (PMC4774998; doi:10.1371/journal.pone.0150364)
Supplement: S3 Fig — (PDF) [file pone.0150364.s003.pdf]

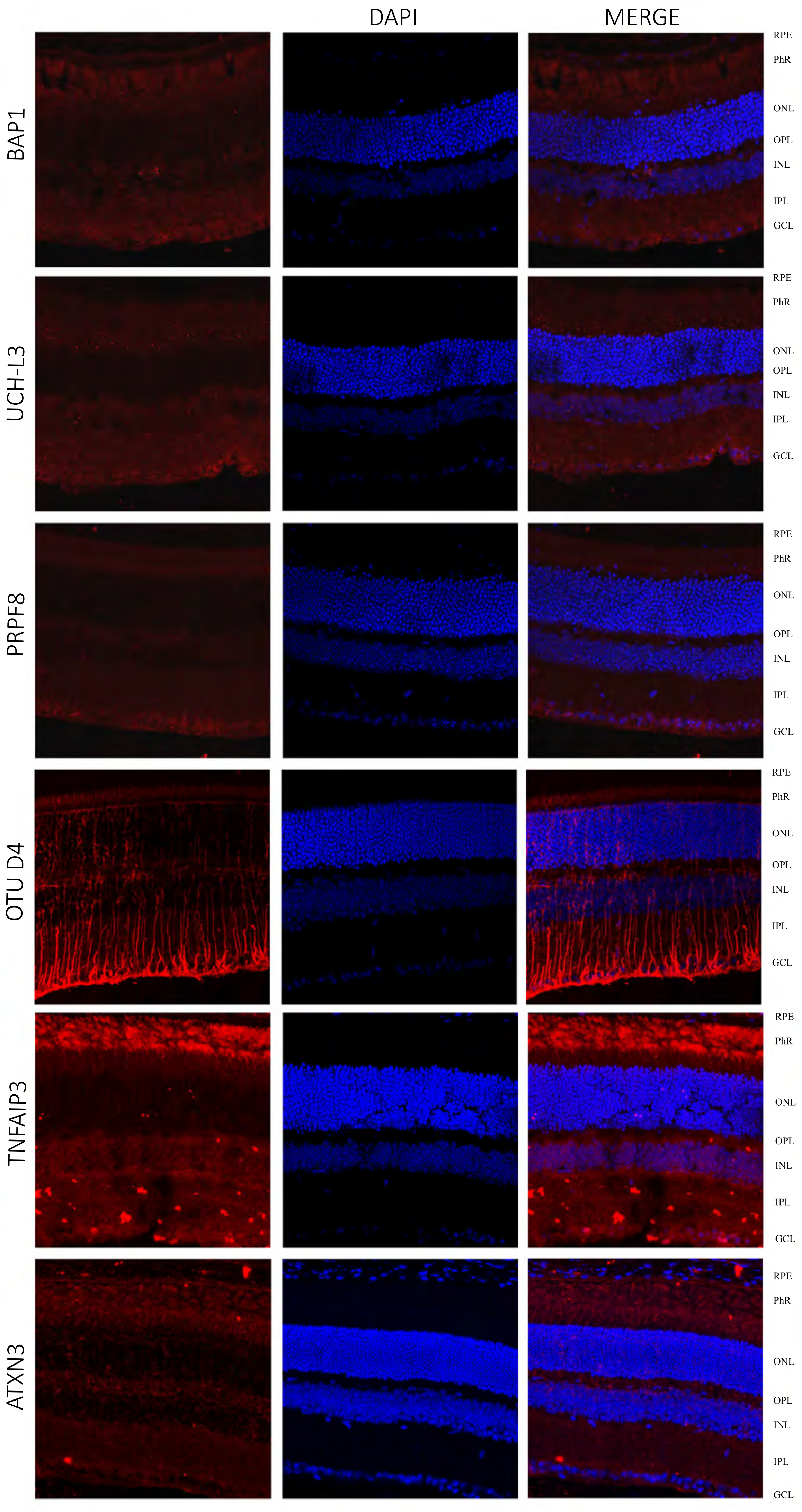
DAPI
MERGE
BAP1
UCH-L3
PRPF8
OTU D4
TNFAIP3
ATXN3
RPE
PhR
ONL
OPL
INL
IPL
GCL

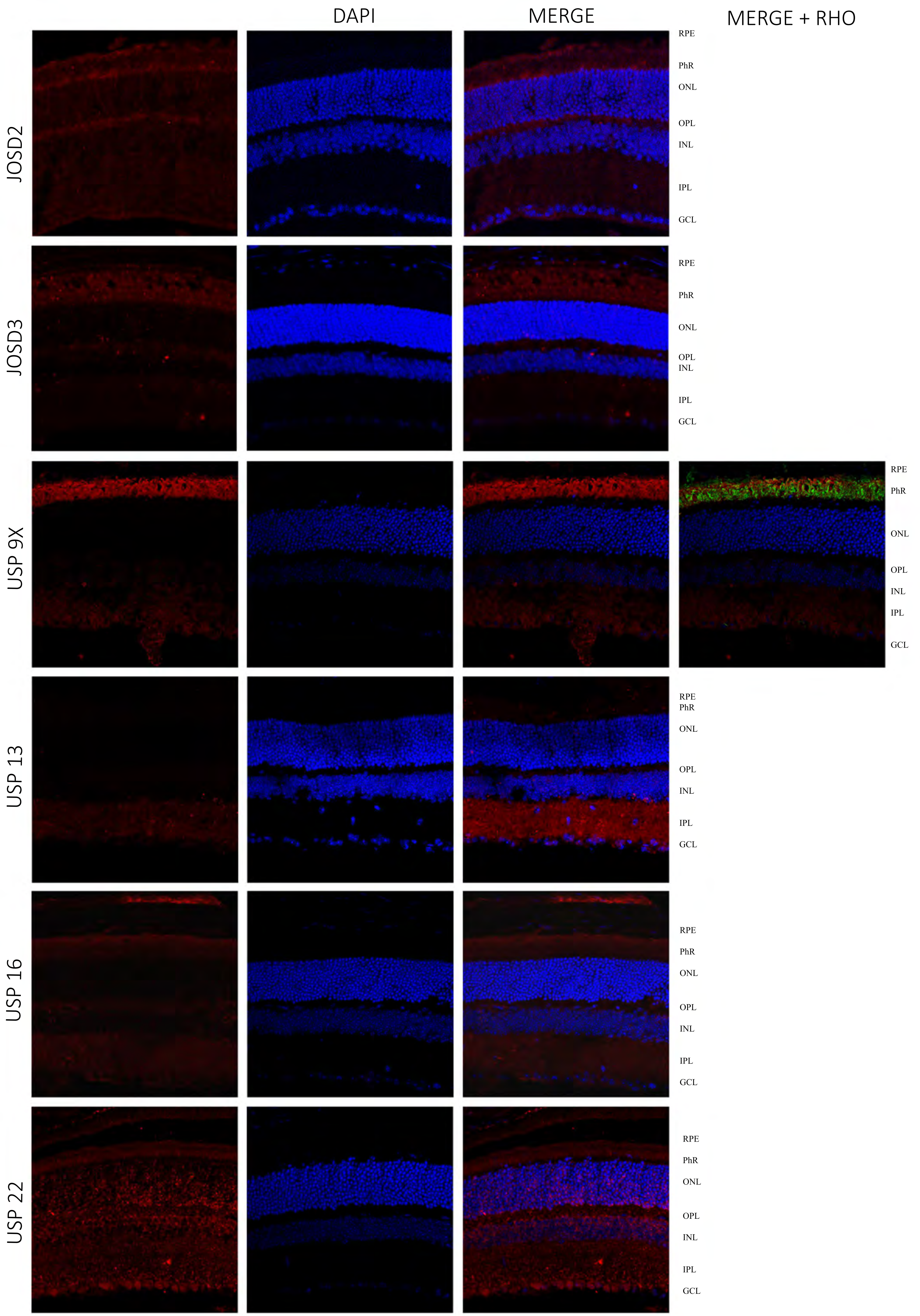
DAPI
MERGE
MERGE + RHO
JOSD2
JOSD3
USP 9X
USP 13
USP 16
USP 22
RPE
PhR
ONL
OPL
INL
IPL
GCL

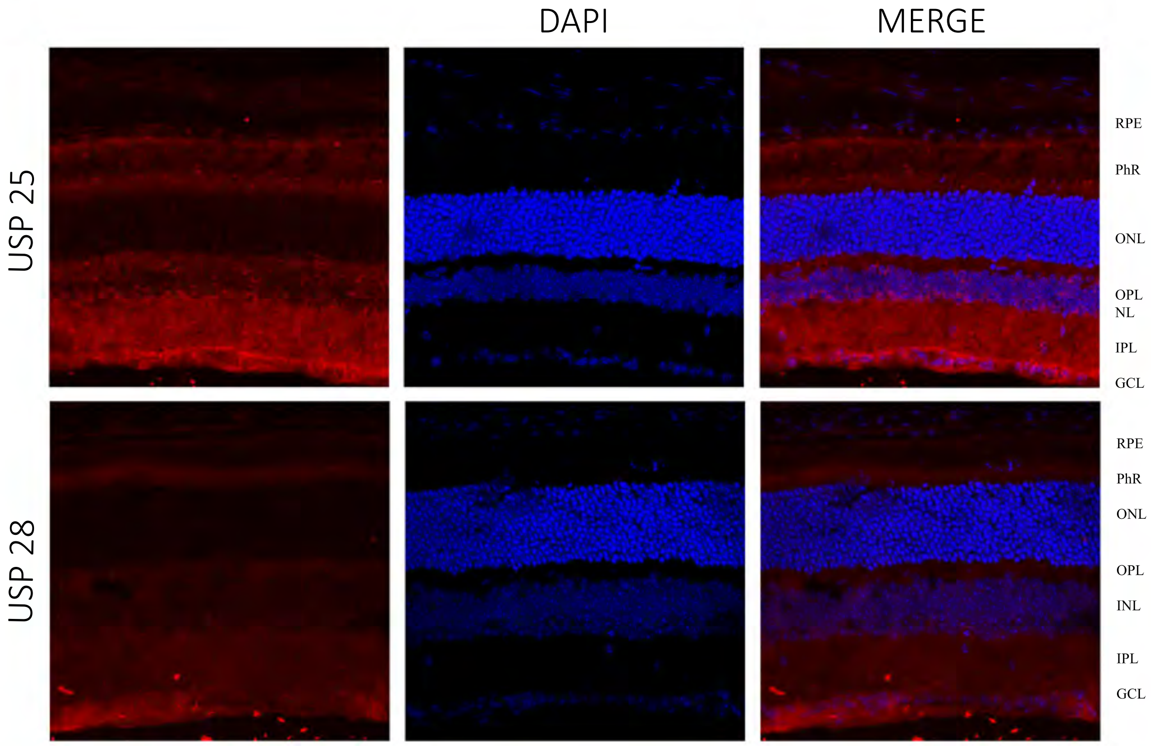

Supplementary Fig 4. Fluorescent immunohistochemistry of selected genes on retinal cryosections, using antibodies against a selection of DUB proteins. The figure shows the immunodetection of the indicated DUBs (in red), DAPI counter-staining of the nuclei (in blue) and the merged channels. In USP9X, the extra image shows its co-localization with Rhodopsin in the outer photoreceptor segment, where phototransduction occurs. **RPE**- Retinal pigmented epithelium; **Phr**- Photoreceptor cell layer; **ONL**- Outer nuclear layer; **OPL**. Outer plexiform layer; **INL**- Inner nuclear layer, **IPL**- Inner plexiform layer; **GCL**- Ganglion cell layer.
