# Supplementary material for: Expression Atlas of the Deubiquitinating Enzymes in the Adult Mouse Retina, Their Evolutionary Diversification and Phenotypic Roles
Source: PLoS One. 2016 Mar 2;11(3):e0150364. doi: 10.1371/journal.pone.0150364 (PMC4774998; doi:10.1371/journal.pone.0150364)

UCH/UCH_1 RAxML

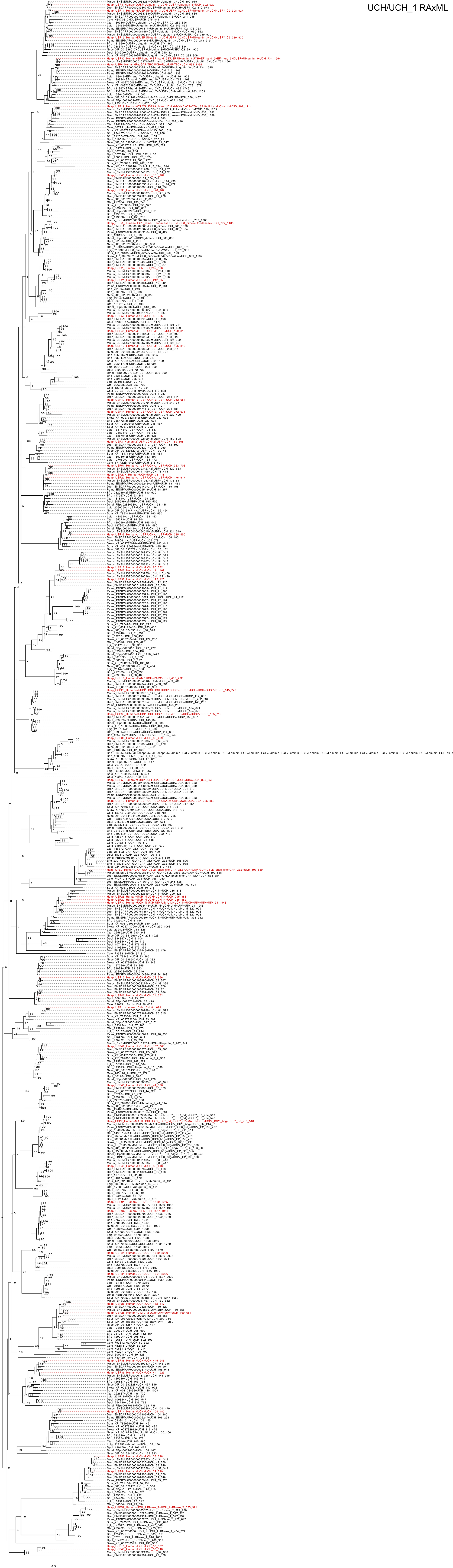

Supplement: S3 File — (ZIP) [file uch.04.bsbt_Arqs.tre.pdf › sm_trees/uch.04.bsbt_Arqs.tre.pdf]
