# Supplementary material for: Expression Atlas of the Deubiquitinating Enzymes in the Adult Mouse Retina, Their Evolutionary Diversification and Phenotypic Roles
Source: PLoS One. 2016 Mar 2;11(3):e0150364. doi: 10.1371/journal.pone.0150364 (PMC4774998; doi:10.1371/journal.pone.0150364)
Supplement: S1 Table — (PDF) [file pone.0150364.s007.pdf]

		Real Time qPCR			In Situ Hybridization	
**Family**	**Gene**	**Orientation**	**Sequence (5'-3')**	**Gene**	**Orientation**	**Sequence (5'-3')**
	Gapdh*	Forward	tgacaatgaatacggctacagcaa			
		Reverse	tactccttggaggccatgtagg			
	Rho	Forward	gcccttctccaacgtcacag	***Rho****	Forward	gcccttctccaacgtcacag
		Reverse	gcagcttcttgtgctgtacgg		Reverse	gcagcttcttgtgctgtacgg
JAMM	***Amsh***	Forward	attgttcaagagttcgggaagg	***Amsh***	Forward	caccgagactacaaatcagctatc
		Reverse	gggggccacatctacacaagg		Reverse	gggggccacatctacacaagg
	Amsh-like	Forward	gctgctatgcctgaccatacaga	***Amsh-like***	Forward	gagaacccagaggcccggacta
		Reverse	gacctgaagtaacggcgtggggtg		Reverse	gacctgaagtaacggcgtggggtg
	Brcc36	Forward	gcggcgttctgacaagagaaagg	***Brcc36***	Forward	aacacaaagactggccgggta
		Reverse	ataccagccaacaactctcatggg		Reverse	cgatgcaggaaccaaagcag
	Csn5	Forward	ttccgggagtggtatggcccag	***Csn5***	Forward	ttccgggagtggtatggcccag
		Reverse	cgccgccaggatttctttcttgttg		Reverse	gtgtactaacatcaatcccggag
	Eif3h	Forward	catgtttgaagaagtgccgattg	***Eif3h***	Forward	NP
		Reverse	gtgcttatccgccacagctgac		Reverse	NP
	Jamm2	Forward	cctgcctgaatgctgtaaagattg	***Jamm2***	Forward	cctgcctgaatgctgtaaagattg
		Reverse	agtaagatggctgccagaattctgt		Reverse	tcctctcttacttctcctctgtg
	Jamm3	Forward	cctgttcctcgaggcctgt	***Jamm3***	Forward	tccatggcagctcccgagtct
		Reverse	tcaatgtcctgcaagcaaggagggta		Reverse	ctcctccaacagcagttcttcc
	Mysm1	Forward	tgtctctgctctgtgccaactg	***Mysm1***	Forward	NP
		Reverse	cacctctcctgctgagaaacca		Reverse	NP
	Poh1	Forward	acatgtggatgtcttatgacttca	***Poh1***	Forward	caatgctaatatgatggtcttgg
		Reverse	gcgccactgacagctctctacgt		Reverse	gccactgacagctctctacg
	Prpf8	Forward	gcgtggaattacccctctgc	***Prpf8***	Forward	NP
		Reverse	cacccgctgcttcgttactg		Reverse	NP
	Psmd7	Forward		***Psmd7***	Forward	NP
		Reverse		***Amsh***	Reverse	NP
MJD	***Atxn***	Forward	gccctgtggagctatcctcaat	***Atxn***	Forward	gccctgtggagctatcctcaat
		Reverse	actcccccttctgccattctc		Reverse	gagggcactctgctctttcaga
	Josd1	Forward	ccatggtctctgcaggtctga	***Josd1***	Forward	ccatagtttgggacagagttggg
		Reverse	tgctcaggattaacatgcaagc		Reverse	tgctcaggattaacatgcaagc
	Josd2	Forward	gtgcgaggtgctattggtggt	***Josd2***	Forward	gccgacgaaatctgcaagag
		Reverse	gcagcagatcagcttgtgttca		Reverse	gcagcagatcagcttgtgttca
	Josd3	Forward	gaacaccaccttaaggaatgcttg	***Josd3***	Forward	ggcatcggatagagctggagat
		Reverse	gtatttgtgcctccgactgtca		Reverse	gtatttgtgcctccgactgtca
OTU	***Otub1***	Forward	agcaggtggacaagcagacctc	***Otub1***	Forward	agcaggtggacaagcagacctc
		Reverse	cagtcgcaggtagaccacaagg		Reverse	cctctgtacatgtctagcgcc
	Otub2	Forward	gccacttaccttgccttgctgc	***Otub2***	Forward	acattctatccattcttcgggatca

		Reverse	acagggtggtcccatggttatc		Reverse	agaagtcagctcggttcctgatg
	Otud1	Forward	catggggcagatgctgaatgtg	***Otud1***	Forward	agaagctagccctgtacctgg
		Reverse	ggcccagatagtggatcatgg		Reverse	ggcccagatagtggatcatgg
	Otud3	Forward	gatgcggaggagaaccatga	***Otud3***	Forward	tccacatcgcctaccgctac
		Reverse	tgccttcatttctgccctga		Reverse	tgccttcatttctgccctga
	Otud4	Forward	cctgttcccgtgtatcctcaga	***Otud4***	Forward	cctgttcccgtgtatcctcaga
		Reverse	catcggtgcaaggacagtcac		Reverse	tccaaaggcaaatccaattctcc
	Otud5	Forward	ctgagcaccctgaactgcat	***Otud5***	Forward	gcctaccgtcatttaagccagg
		Reverse	tgaaggaggtttggcaagagcta		Reverse	tgaaggaggtttggcaagagcta
	Otud6a	Forward	cgagatggagcagaggcacaa	***Otud6a***	Forward	NP
		Reverse	gctgtaaccgaatccacactgc		Reverse	NP
	Otud6b	Forward	ccgggaagaaaggatagcagagg	***Otud6b***	Forward	NP
		Reverse	ggctgccaatatttgagcaagtt		Reverse	NP
	Otud7a	Forward	tctgacggattcggaacacaag	***Otud7a***	Forward	cttgcacagccagagtctcc
		Reverse	tggctaaccgggcattgtcg		Reverse	ggcgacgctcccgacgc
	Otud7b	Forward	atgatccagcgttaccttgcag	***Otud7b***	Forward	gggcctgatgcacagcaagg
		Reverse	ccctccattcatgatcttcctc		Reverse	ccctccattcatgatcttcctc
	Parp11	Forward	tgctatccccatccagctttg	***Parp11***	Forward	NP
		Reverse	tgcttcccaatttgagtgactg		Reverse	NP
	Tnfaip3	Forward	ataatggattctgtgagcgttgc	***Tnfaip3***	Forward	tttgagcttgttcagcacgaatac
		Reverse	gaaggcaggcttggcactttc		Reverse	gaaggcaggcttggcactttc
	Vcpip1	Forward	atggggtgtgcctcaggaccttatt	***Vcip1***	Forward	gaaacgaagggaccgaagaatc
		Reverse	ctgcaaacttctgtctcctccaa		Reverse	tgctgctgaaagtgctgctta
	Yod1	Forward	cagcgtaacttccctgatccaga	***Yod1***	Forward	gaaggaccgaggccgagtc
		Reverse	cttcatcagctaattccagtgcttg		Reverse	cgggtagccaacgaggattc
	Zranb1	Forward	tcccagacctaataacattgaagca	***Zranb1***	Forward	ggtggaagtagtcctttgatatg
		Reverse	ctccatcgagctctgtcttgctc		Reverse	ctccatcgagctctgtcttgctc
UCH	***Bap1***	Forward	tcctgggagtgggagtgacat	***Bap1***	Forward	ctgcttcctgagcattgaggag
		Reverse	ctgcttcctgagcattgaggag		Reverse	gctgtgactcttgagatttgtg
	Uch-l1	Forward	gattaaccccgagatgctgaac	***Uch-l1***	Forward	cgcttcgccgacgtgctagg
		Reverse	ggatggcactgagcccagag		Reverse	gcccatcgagctcgtacaga
	Uch-l3	Forward	tatgcgcgcagtgttactcctctt	***Uch-l3***	Forward	tatgcgcgcagtgttactcctctt
		Reverse	tcgttccacaggcattgctgat		Reverse	ggcatcctctaacaacgtctcatc
	Uch-l5	Forward	agatgtgattcgacaagtgcacaa	***Uch-l5***	Forward	ggggtcttcaccgagctcat
		Reverse	agatgtgattcgacaagtgcacaa		Reverse	agatgtgattcgacaagtgcacaa
USP	***Usp1***	Forward	ggacaccattcaccgacaatag	***Usp1***	Forward	NP
		Reverse	aacgcctgtttggtcactggat		Reverse	NP
	Usp2	Forward	acctgaagcgattctcagaatc	***Usp2***	Forward	gatggtctccacaatgaggtg
		Reverse	ctctcaagtccaggtctctta		Reverse	ctctcaagtccaggtctctta
	Usp3	Forward	ccgctggttccatttcaatgac	***Usp3***	Forward	NP

	Reverse	ctggcctgacgctccacataa		Reverse	NP
Usp4	Forward	tggtgaggacgatcagggaga	***Usp4***	Forward	NP
	Reverse	aggagttcacgaggctgaagg		Reverse	NP
Usp5	Forward	cacccatgctggacgaatccg	***Usp5***	Forward	cggatgagcccaaaggtagcct
	Reverse	gccccgctgtttcccgtatag		Reverse	gccccgctgtttcccgtatag
Usp6	Forward	cggaagtatctggagctgagc	***Usp6***	Forward	NP
	Reverse	cagtccatctttcggcccttc		Reverse	NP
Usp7	Forward	gatgatgacgtggtatccaggtg	***Usp7***	Forward	NP
	Reverse	catataggcatttgtgcagtgtc		Reverse	NP
Usp8	Forward	caacgagcacctggatgacc	***Usp8***	Forward	tatttggaggatcaggaccagc
	Reverse	acgatgatggactcgttgagc		Reverse	acgatgatggactcgttgagc
Usp9X	Forward	ttaaaaggaaatggacctgggc	***Usp9X***	Forward	tcaagaatgcagtcttcgatca
	Reverse	gctttgcactggaggagacc		Reverse	gctttgcactggaggagacc
Usp9Y	Forward	gtggacttgggctatggaatgg	***Usp9Y***	Forward	NP
	Reverse	gtactggaggagaccagttgc		Reverse	NP
Usp10	Forward	atgcccaggacttgtgacagc	***Usp10***	Forward	NP
	Reverse	cctggcatcgcctcctagtg		Reverse	NP
Usp11	Forward	gcctggcagaaccataaacgac	***Usp11***	Forward	NP
	Reverse	cattgccacaatcagggcacac		Reverse	NP
Usp12	Forward	accagcttcaccggtacacga	***Usp12***	Forward	NP
	Reverse	ctgtcagggttggtcgcatct		Reverse	NP
Usp13	Forward	cactggactggatcttcagcc	***Usp13***	Forward	tgatgaaccagttgatagaccc
	Reverse	gcctcagacacgatgttggca		Reverse	gcctcagacacgatgttggca
Usp14	Forward	atggaattgccatgtggattgac	***Usp14***	Forward	NP
	Reverse	gggcatctttgagttcaggcac		Reverse	NP
Usp15	Forward	cgcgcagtcacttaaggagca	***Usp15***	Forward	NP
	Reverse	ccatcagtgtgtaccagctgac		Reverse	NP
Usp16	Forward	agcacttgcgggagaagtgga	***Usp16***	Forward	aagggttggaacaaggtaatttg
	Reverse	ggccatccaaatccttctggtta		Reverse	caggttttccgatttctctctct
Usp17L8	Forward	ctgccaatgacaagcccagtc	***Usp17L8***	Forward	NP
	Reverse	cagggctgcattcaggtagca		Reverse	NP
Usp18	Forward	acgcaggagtccctgatttgc	***Usp18***	Forward	NP
	Reverse	cagaggctttgcgtccttatcaa		Reverse	NP
Usp19	Forward	cctggctctggtgtggcgga	***Usp19***	Forward	NP
	Reverse	cagcagagcctggatcttcagc		Reverse	NP
Usp20	Forward	cggcatgaggtgatgtactcct	***Usp20***	Forward	tggctggccttcatcgtgg
	Reverse	cagggacgtgcactccttgg		Reverse	cagggacgtgcactccttgg
Usp21	Forward	ccgagtgggagccaagatacc	***Usp21***	Forward	NP
	Reverse	cctgggaggcaaaggtcgtaa		Reverse	NP
Usp22	Forward	tctaccccattctggcccttg	***Usp22***	Forward	NP

	Reverse	ttcgcaggcagtctgtgagag		Reverse	NP
Usp24	Forward	gtgtgcccagacaggatgctc	***Usp24***	Forward	NP
	Reverse	ggttctcctcccactcctgg		Reverse	NP
Usp25	Forward	agagcagccatcaagaagtgac	***Usp25***	Forward	ccaccgggagagccggtggat
	Reverse	atcttcaagatcatgtgacgcct		Reverse	atcttcaagatcatgtgacgcct
Usp26	Forward	cgcaaggtggatccaacaaagt	***Usp26***	Forward	NP
	Reverse	tagtggcctccattgggactg		Reverse	NP
Usp27X	Forward	tctggacttgcctggctcttg	***Usp27X***	Forward	NP
	Reverse	agtggtgatgcctgggatgtg		Reverse	NP
Usp28	Forward	catgaggagtactccaggctct	***Usp28***	Forward	catgaggagtactccaggctct
	Reverse	catgctgaaggcgagggtcac		Reverse	acaccaggtaggacaatgcttc
Usp29	Forward	ccagcgcagaagtgaacaagg	***Usp29***	Forward	NP
	Reverse	ttctctgctttgccgcctctg		Reverse	NP
Usp30	Forward	ctgccacagtgcctctgcat	***Usp30***	Forward	actggaagtctcagcaccct
	Reverse	aactgcacgtgctcgtgcc		Reverse	aactgcacgtgctcgtgcc
Usp31	Forward	ctgacagagccagcgtcacct	***Usp31***	Forward	NP
	Reverse	acgtgcttgccatccactctg		Reverse	NP
Usp32	Forward	caccgactctgcctacattctt	***Usp32***	Forward	NP
	Reverse	ctcgtgtccgccatcttcttg		Reverse	NP
Usp33	Forward	tcctccgacctccagttgttc	***Usp33***	Forward	NP
	Reverse	gtgtcctcagaactacaaagagc		Reverse	NP
Usp34	Forward	atggcaggtttgacgactgt	***Usp34***	Forward	tgacgaaggagcaactcctgt
	Reverse	tcggattcatcttcagctagtg		Reverse	tcggattcatcttcagctagtg
Usp35	Forward	catggtggcctctctggtcaa	***Usp35***	Forward	NP
	Reverse	agcccgggaaccgaaacacc		Reverse	NP
Usp36	Forward	gcatcgacacgctcctccca	***Usp36***	Forward	gcatcgacacgctcctccca
	Reverse	gcaccctcaccctccgagc		Reverse	ctcttcatcctgctgtggctc
Usp37	Forward	agaggtgctggcagctgtgtt	***Usp37***	Forward	NP
	Reverse	gtgtccggactgctggttgg		Reverse	NP
Usp38	Forward	cagcttcgttacccagtgctca	***Usp38***	Forward	NP
	Reverse	gacaaaggcagctgttgctgga		Reverse	NP
Usp39	Forward	caagccgtccacaagaacacc	***Usp39***	Forward	NP
	Reverse	tgatgaagcacgtggatcctg		Reverse	NP
Usp40	Forward	ctgaggacacagctacgcatc	***Usp40***	Forward	NP
	Reverse	tcaaggagccagcagtccatc		Reverse	NP
Usp41	Forward		***Usp41***	Forward	NP
	Reverse			Reverse	NP
Usp42	Forward	acaacgtcgacttcccccagt	***Usp42***	Forward	NP
	Reverse	tctgaggactcggccccatag		Reverse	NP
Usp43	Forward	gactcgggagcctcaacaaca	***Usp43***	Forward	NP

	Reverse	tggtgaagacaggagagtcgg		Reverse	NP
Usp44	Forward	gagcccagttccccgtacag	***Usp44***	Forward	NP
	Reverse	agcatggcaaacggtgagacc		Reverse	NP
Usp45	Forward	agcctcactgacggcagcg	***Usp45***	Forward	agcctcactgacggcagcg
	Reverse	aggctgcttggaagcgatc		Reverse	gacaggactggactgagcat
Usp46	Forward	accgggtggtcttccctctg	***Usp46***	Forward	NP
	Reverse	aacgaccacagcaaccaggtc		Reverse	NP
Usp47	Forward	gtccatgtcacagcttgccatc	***Usp47***	Forward	gagagtacagagttaaagtgtgcc
	Reverse	gctttccaacacaagctcctgg		Reverse	gctttccaacacaagctcctgg
Usp48	Forward	gcagcagcaggatgcacaaga	***Usp48***	Forward	NP
	Reverse	ctgctgcacaacattccgaaca		Reverse	NP
Usp49	Forward	caggagcctggagctcattca	***Usp49***	Forward	NP
	Reverse	cccacttcccagaccacatga		Reverse	NP
Usp50	Forward	actttggagatctggatggtgg	***Usp50***	Forward	NP
	Reverse	tcactgactcgggtgtcatcaa		Reverse	NP
Usp51	Forward	gacctgggtagcagtgccaaa	***Usp51***	Forward	NP
	Reverse	gaaagcaggccacaatcggtaa		Reverse	NP
Usp52	Forward	cggaagtatctggagctgagc	***Usp52***	Forward	NP
	Reverse	cagtccatctttcggcccttc		Reverse	NP
Usp53	Forward	ggagtccatgcatgacccagg	***Usp53***	Forward	tgaacaactggacgggtagctg
	Reverse	tgaacaactggacgggtagctg		Reverse	catctgtgagaactgctgggct
Usp54	Forward	cacgtgcaacctgcctagaa	***Usp54***	Forward	gaagagagcactgtcgctgg
	Reverse	tccagttcagaggtctcctgc		Reverse	tccagttcagaggtctcctgc
Cyld	Forward	tcccaggcagtgccgcatc	***Cyld***	Forward	tcccaggcagtgccgcatc
	Reverse	tgcttgatcttcccagctgag		Reverse	gaatgttgaagccattctgacc

Supplementary Table 1.- Sequences of the primer pairs used in the Reverse Transcriptase Real Time qPCR (left) and *in situ* hybridisation (right) including all the DUB families.

* *Gapdh*, *Rho* and *Cerkl* genes were used for normalization and reference high and low expression values, respectively.

** Genes whose *in situ* hybridisation did not yield a reproducible pattern.

NP - cases where *in situ* hybridisation was not performed, given the low expression levels detected in the RT-qPCR.
