# Supplementary material for: Expression Atlas of the Deubiquitinating Enzymes in the Adult Mouse Retina, Their Evolutionary Diversification and Phenotypic Roles
Source: PLoS One. 2016 Mar 2;11(3):e0150364. doi: 10.1371/journal.pone.0150364 (PMC4774998; doi:10.1371/journal.pone.0150364)
Supplement: S2 Table — (PDF) [file pone.0150364.s008.pdf]

Supplementary Table 2.- Mutant neuronal and retinal phenotypes in different animal models and human listed per DUB family and gene.

ND- Not determined (The list of bibliography is included at the end)

JAMM								
	D. melanogaster [1]		***D. rerio*** [2]		***M. musculus***		***H. sapiens***	
	**Neuronal phenotype**	**Retinal phenotype**	**Neuronal phenotype**	**Retinal phenotype**	**Neuronal phenotype**	**Retinal phenotype**	**Neuronal phenotype**	**Retinal phenotype**
**AMSH**	ND	ND			Loss of neurons in the hippocampus and cerebral cortex [3]		Microcephaly-capillary malformation syndrome (OMIM)	
**AMSH-Like**								
**BRCC36**	ND	ND						
**CSN5**					-/- homozygotes die soon after implantation and exhibit growth-retardation, decrease in cell proliferation, and an increase in cell apoptosis.			
**CSN6**	Pupal death		1dpf: **cns necrosis**, fused somite; 4dpf: small size and short body, small head, pericardial edema, unconsumed egg yolk with no yolk extension, **shorten notochord length**, dysmorphic axis, underdeveloped tail	4dpf: small eyes	-/- homozygotes are embryonic lethal [4]			
**EIF3H**	Pan-neural knockdown is developmentally lethal							
**JAMM2**								
**JAMM3**								
**MYSM1**	ND	ND	1dpf: **cns necrosis**, curled tail developed; 3dpf: narrow head; closed otoliths, abnormal yolk shape with short yolk extension, **abnormal notochord shape**, slightly curled tail, ratty caudal fin	3dpf: funny eyes shape				
**POH1**	Larval death							
**PRPF8**						Retinal degeneration in heterozygotes, more severe in homozygotes [4]		autosomal dominant retinitis pigmentosa
**PSMD7**			1dpf: **serious cns necrosis**; 2dpf:		-/- homozygotes are embryonic			

Supplementary Table 2.- Mutant neuronal and retinal phenotypes in different animal models and human listed per DUB family and gene.

ND- Not determined (The list of bibliography is included at the end)

			small and short body, underdeveloped head, preicardical edema, **shorten notochord**, thin trunk, fused somite, shorten and fatty tail, shorten yolk extension		lethal [4]			

MJD								
	D. melanogaster		*D. rerio*		*M. musculus*		*H. sapiens*	
	**Neuronal phenotype**	**Retinal phenotype**	**Neuronal phenotype**	**Retinal phenotype**	**Neuronal phenotype**	**Retinal phenotype**	**Neuronal phenotype**	**Retinal phenotype**
**ATX3**	ND	ND	1dpf: **cns necrosis**, fused somite; 3dpf: reduced body length, dysmorphic axis development, malformed and shorter tail				Neurodegenerative disease SCA3 [5]	
**JOSD1**	ND	ND	1dpf: **serious cns necrosis**, slightly trunk and tail necrosis, fused somite, shorten tail; 3dpf: small head, strange yolk shape**, abnormal notochord shape**, irregular floorplate, hemorrhage at tail region, fused somite, shorten and curled tail	3dpf: funny eyes shape, abnormal retinotectal projection				
**JOSD2**			1dpf: abnormal head shape, fused somite; 4dpf: small and short body, strange head shape, tectum enlarged, pericardial edema, unconsumed egg yolk, fat and short yolk extension, dysmorphic axis development, **abnormal notochord shape**, curled tail					
**JOSD3**	ND	ND	ND	ND				

OTU								
	D. melanogaster		*D. rerio*		*M. musculus*		*H. sapiens*	
	**Neuronal phenotype**	**Retinal phenotype**	**Neuronal phenotype**	**Retinal phenotype**	**Neuronal phenotype**	**Retinal phenotype**	**Neuronal phenotype**	**Retinal phenotype**
**OTUB1**			1dpf: **cns necrosis**, fused somite; 2dpf: small and short body, round					

Supplementary Table 2.- Mutant neuronal and retinal phenotypes in different animal models and human listed per DUB family and gene.

ND- Not determined (The list of bibliography is included at the end)

			yolk shape with thin yolk extension, thin trunk, curled tail			
**OTUB2**	ND	ND	ND	ND		
**OTUD1**	ND	ND	ND	ND		
**OTUD3**	ND	ND	1dpf: **slightly cns and trunk necrosis**, fused somite; 3dpf: **serious dysmorphic development of axis and notochord**, curled tail			
**OTUD4**			1dpf: underdeveloped trunk; 3dpf: small and short body, reduced pigementation, **cns necrosis**, dysmorphic notochord shape, thin trunk, slightly curled tail (Tse)	3dpf: undeveloped eye (Tse). Reduction in size of the optic tecta and cerebellum (Margolin 2013)		Mutated, together with RNF216, in cerebellar ataxia and hypogonadotropic hypogonadism (Gordon Holmes syndrome) [6]
**OTUD5**			1dpf: **cns necrosis**, fused somite; 3dpf: reduced pigement, small and short body, pericardial edema, thin and short yolk extension, slightly bent body		Abnormal embryo turning and developmental patterning [4]	
**OTUD6a**	ND	ND	ND	ND		
**OTUD6b**						
**OTUD7a**	ND	ND				
**OTUD7b**	ND	ND	ND<br>1dpf**: underdeveloped trunk and notochord**, fused somite; 3dpf: small and short body, slightly trunk necrosis, fused and curled tail	ND		
**PARPF11**			1dpf: **cns necrosis**, fused somite; 4dpf: strange head shape, small and short body, dysmorphic axis shape, thin trunk curled tail, **ratty caudal fin**			
**TNFAIP3**	ND	ND	1dpf: trunk and tail necrosis; 4dpf: abnormal head shape, pericardial edema, **axis and notochord problem**s, thin trunk, unconsumed egg yolk with no yolk extension, short and curled tail			
**VCPIP1**	ND	ND	1dpf: **underdeveloped trunk and notochord**, shorten and fused tail;	4dpf: small eyes		

Supplementary Table 2.- Mutant neuronal and retinal phenotypes in different animal models and human listed per DUB family and gene.

ND- Not determined (The list of bibliography is included at the end)

			4dpf: small and small body, small head, thin trunk, abnormal somite shape, curled and thin tail					
**YOD1**			No early phenotype; 3dpf: short and edema body, pericardial edema, unconsumed egg yolk with fat and shorten yolk extension, underdeveloped liver and gut, **abnormal axis and notochord** shape, fused somite, short and curled tail					
**ZRANB1**	Pharate adult and young adult death (Sokol)		1dpf:fused somite, curled tail developed; 3dpf: shorten body length, **abnormal notochord shape**, no yolk extension, curled tail					

**UCH**								
	D. melanogaster		***D. rerio***		***M. musculus***		***H. sapiens***	
	**Neuronal phenotype**	**Retinal phenotype**	**Neuronal phenotype**	**Retinal phenotype**	**Neuronal phenotype**	**Retinal phenotype**	**Neuronal phenotype**	**Retinal phenotype**
**BAP1**			1dpf: **serious cns and trunk necrosis**, fused somite; 4dpf: hemorrhage in head, unconsumed egg yolk, **abnormal notochord shape**, curled tail		-/- homozygotes are embryonic lethal(Clague)			
**UCHL1**	ND	ND			Gracile axonal dystrophy [5]		Linked to PD and other diseases [5]Childhood-Onset Neurodegeneration With Optic Atrophy: Progressive visual loss due to optic atrophy at around age 5 years, followed by spasticity, cerebellar ataxia, peripheral neuropathy, and myokymia, consistent with systemic neurodegeneration and deficits at the neuromuscular junction (OMIM)	
**UCHL3**			1dpf: slightly trunk necrosis, fused somite; 3dpf: small and short body, pericardial edema, fat and short yolk	3dpf: reduced eye pigment	Learning and working memory deficits [5]	Retinal degeneration [5]		

Supplementary Table 2.- Mutant neuronal and retinal phenotypes in different animal models and human listed per DUB family and gene.

ND- Not determined (The list of bibliography is included at the end)

			extension, **abnormal notochord shape**, curled and malformed tail					
**UCHL5**	Pupal and pharate adult death (FUNC: NEUROGENESIS)		1dpf: **cns necrosis**, abnormal head shape, fused somite; 4dpf: thick looking jaw, enlarged otolith, pericardial edema, thin yolk extension, slightly body bent and curled tail, ratty caudal fin		Prenatal lethality, severely abnormal brain development [4]			

**USP**								
	D. melanogaster		***D. rerio***		***M. musculus***		***H. sapiens***	
	**Neuronal phenotype**	**Retinal phenotype**	**Neuronal phenotype**	**Retinal phenotype**	**Neuronal phenotype**	**Retinal phenotype**	**Neuronal phenotype**	**Retinal phenotype**
**USP 1**	(Neuronal) Slower Adults		No early phenotype; 3dpf: **abnormal notochord development**, body bent, thin trunk and curved tail		Elevated perinatal lethality, male infertility. Fancony anemia [4]			
**USP 2**	Pan-neuronal knockdown leads to reduced locomotion and earlier adult death [5]							Upregulated in high-grade gliomas [5]
**USP 3**	ND	ND	1dpf: **serious cns necrosis**, fused somite; 2dpf: small and short body, underdeveloped head, reduced pigmentation, enlarged tectum, thin trunk, body bent**, abnormal notochord development**, curled and fused tail	2pdf: underdeveloped eyes				
**USP 4**	ND	ND	ND<br>1dpf: **cns necrosis**; 2dpf: small and short body, small head, unconsumed yolk with thin extension, notochord problem, curled tail	ND<br>2dpf: small eyes				
**USP 5**	Pupal death(Sokol)		1dpf: **slightly cns necrosis**, undeveloped trunk, fused tail; 2dpf: small and short body, abnormal head shape, percardial edema, dysmorphic axis shape, shorten notochord, trunk and tail necrosis,	2dpf: underdeveloped small eyes	-/- homozygotes are embryonic lethal [4]			

Supplementary Table 2.- Mutant neuronal and retinal phenotypes in different animal models and human listed per DUB family and gene.

ND- Not determined (The list of bibliography is included at the end)

			short and curled tail			
**USP 6**	ND	ND	ND	ND		
**USP 7**	Pharate adult death		1dpf: **slightly cns damage**; 2dpf: small and short body, small head, reduced pigment, "bud" on head, **abnormal notochord development**, fused tail, ratty caudal fin, ; 3dpf: close otoliths, slightly percardial edema, unconsumed yolk in pear shape	2dpf: small eyes	-/- homozygotes are embryonic lethal(Clague). Brain specific knockdown causes brain malformation and neonatal lethality, due at least in part to p53-dependent mechanisms. [5]	
**USP 8**	Larval death		1dpf: **serious cns and truck necrosis**, fused somite; 3dpf: small body, **slightly cns necrosis**, percardial edema, **abnormal notochord development**, fused somite, curled tail with ratty caudal fin		Brain development deficiencies in brain targeted knockout (Hausp fl/fl nes-Cre) mice	
**USP 9X**	Fat facets		ND	ND	reduction in axonal length and arborisation, decrease in neuronal migration [7]	Involved in neuronal fate specification and NMJ function.(Sokol?) patients with multiple myeloma overexpressing USP9X have a poor prognosis [8]
**USP 9Y**	ND	ND	ND	ND		
**USP 10**			1dpf: **cns necrosis**, fused somite; 2dpf: small and short body, reduced pigment, thin trunk, body bent ventrically, curled tail, ratty caudal fin			
**USP 11**	ND	ND				
**USP 12**	ND	ND	1dpf**: cns and tail necrosis**; 4dpf: slightly edema body, pericardial edema, unconsumed yolk, abnormal yolk shape with thin yolk extension, **dysmorphic notochord and axis shape**, curled tail			
**USP 13**	ND	ND	1dpf: **cns necrosis**, fused somite; 2dpf: small and short body, reduced pigment, thin trunk, **dysmorphic notochord shape**, pericardial edema, slightly curled tail			
**USP 14**	(Neuronal)Slower				Mutations in intron leads to	

	adults, early death (Sokol)				redyced USP14 leves. Tremors, abnormal brain morphology, altered synaptic transmission and increased apoptosis. [4]	
**USP 15**	ND	ND	1dpf: **cns necrosis**, fused somite; 2dpf: **underdeveloped head and trunk, axis and notochord problems**, pericardial edema, fused somite, short and slightly curled tail; 4dpf: unshaped head; body edema, round shape unconsumed yolk, underdeveloped liver and gut, tail slightly necrosis	2dpf: small eyes 4dpf: unshaped eyes		
**USP 16**	ND	ND			reduces the self-renewal of hematopoietic stem cells and the expansion of mammary epithelial cells, neuroprogenitors, and fibroblasts [9]	overexpression of USP16 reduces the expansion of normal fibroblasts and postnatal neural progenitors, whereas downregulation of USP16 partially rescues the proliferation defects of Down syndrome [9]
**USP 17**	ND	ND	ND	ND		
**USP 18**	ND	ND	1dpf: **cns and tail necrosis**, fused somite; 2dpf: underdeveloped trunk, tail necrosis; 3dpf: small and short body, small head, thin trunk, **shorten notochord length**, serious pericardial edema, unconsumed yolk with short yolk extensionshorten, short and curved tail	3dpf: small eyes	Tremors, seizures, abnormal nervous system, death [4]	
**USP 19**	ND	ND	1dpf: **slightly cns necrosis**, fused somite; 2dpf: small and short body, reduced pigement, small head, **shorten notochord length**, pericardial edema, short and curled tail with slightly necrosis (with "buds"),	2dpf: small eyes		
**USP 20**	Earlier adulth death		1dpf: **cns necrosis**, fused somite; 2dpf: small and short body, reduced pigment, "buds" on the pericardial and yolk area, tail necrosis; 3dpf: small head, precardial edema, "buds" (necrosis) on the precardical	3dpf: small eyes		

Supplementary Table 2.- Mutant neuronal and retinal phenotypes in different animal models and human listed per DUB family and gene.

ND- Not determined (The list of bibliography is included at the end)

			area, unconsumed yolk with short extension, curled tail with slightly necrosis at the end			
**USP 21**	ND	ND	1dpf: mild necrosis throughout the body; 3dpf: underdeveloped head, small head, **abnormal axis and notochord development**, trunk necrosis, pericardial edema, roundly and unconsumed yolk with no yolk extension, no tail	3dpf: small eyes		
**USP 22**		Axonal projection of photoreceptor cells [5]	No early phenotype at 1dpf; 2dpf: inflated brain		-/- homozygotes are embryonic lethal [4]	
**USP 24**	ND	ND	1dpf: body necrosis, fused somite; 2dpf: small and short body, **cns necrosis became more seriously**, **abnormal notochord development**, thin trunk, fat and short yolk extension, curled short tail with necrosis	2dpf: abnormal eyes		May be involved in PD susceptibility [5]
**USP 25**	ND	ND	1dpf**: cns and tail necrosis**; 2dpf: small and short body, unshaped head, **cns necrosis**, bulging forebrain, thin trunk, fused somite, tail necrosis	2dpf: unshaped eyes		Overexpressed in Down Syndrome brains [5]
**USP 26**	ND	ND	ND	ND		
**USP 27**	ND	ND	ND	ND		
**USP 28**	ND	ND	ND<br>1dpf: head damage, fused somite; 2dpf: **serious cns necrosis**, reduced pigment, **notochord and axis underdevelopment**, thin trunk, shorten and curved tail	ND		
**USP 29**	ND	ND	ND	ND		
**USP 30**	Knockdown of Usp30 in dopaminergic neurons protects flies against paraquat toxicity in vivo, ameliorating defects in					

Supplementary Table 2.- Mutant neuronal and retinal phenotypes in different animal models and human listed per DUB family and gene.
ND- Not determined (The list of bibliography is included at the end)

	dopamine levels, motor function, and organismal survival. [10]					
**USP 31**	ND	ND				
**USP 32**						
**USP 33**	ND	ND	1dpf: **cns necrosis**; 3dpf: small and short body, small head, **notochord development problem**, curled tail	3dpf: small eyes	in cultured mouse embryonic commissural axons and was required for growth cone collapse in response to Slit exposure [11]	Regulates axonal pathfinding during development [5] (AQUI??))
**USP 34**	Pan neuronal knockdown is developmentaly lethal [5]					
**USP 35**			ND	ND		
**USP 36**	Larval death		1dpf**: slightly cns necrosis**; 3dpf: small and short body, reduced pigment, very thin trunk, pericardial edema, funny yolk shape with fat yolk extension, curled tail			
**USP 37**	ND	ND	1dpf: **cns necrosis**; 3dpf: small and short body, small head, tectum enlargement, pericardial edema, round yolk shape, thin trunk, **notochord problem**, shorten and slightly curled tail	3dpf: small eyes		
**USP 38**	ND	ND				
**USP 39**	Larval death		1dpf: **cns and tail necrosis**, 2dpf: small and short body, small head, slightly pericardial edema, dysmorphic notochord development, fused somite, curled tail	2dpf: small eyes		
**USP 40**	ND	ND				May be involved in PD susceptibility [5]
**USP 41**	ND	ND	ND	ND		
**USP 42**	ND	ND	1dpf: **slightly cns necrosis**, fused somite; 3dpf: inflated hindbrain, slightly pericardial edema and body bent			
**USP 43**			1dpf: **slightly cns necrosis**, fused somite; 2dpf: small and short body,	2dpf: small eyes		

Supplementary Table 2.- Mutant neuronal and retinal phenotypes in different animal models and human listed per DUB family and gene.

ND- Not determined (The list of bibliography is included at the end)

			reduced pigment, small head, **bulging forebrain**, pericardial edema, curled tail			
**USP 44**	ND	ND	1dpf: **abnormal development, cns and tail necrosis**, fused somite; 3dpf: very small and short body, underdeveloped head and trunk, **shorten notochord length**, axis development problem, serious pericardial edema, unconsumed round yolk with short yolk extension, short and curled tail			
**USP 45**	Pupal, pharate adult and young adult death		1dpf: **cns necrosis**; 2dpf: reduced pigment, a bit smaller head, close otolith distance, slightly pericardial edema, thin yolk extension	2dpf: reduced eyes		
**USP 46**					role in the GABAergic neurotransmission [12]	
**USP 47**						
**USP 48**	ND	ND	No early phenotype; 3dpf: small and short body, reduced pigement, small head, pericardial edema, body bent, slightly tail necrosis	3dpf: small eyes		
**USP 49**	ND	ND	ND	ND		
**USP 50**	ND	ND	ND	ND		
**USP 51**	ND	ND	ND	ND		
**USP 52**			ND	ND		
**USP 53**	ND	ND	No early phenotype at 1dpf; 3dpf: small and short body, reduced pigment, small head**, inflated brain**, **notochord problem**, curled tail	3dpf: small eyes		
**USP 54**	Pharate adult and young adult death					
**CYLD**			1dpf: **cns necrosis**; 2dpf: small and short body, strange head shape, **thinner mid/hidbrain boundary**, **midbrain enlargement**, round yolk shape with thin extension, slightly axis and notochord problem, curled tail<br><br>1dpf: **cns necrosis**, trunk necrosis;	1dpf: small eyes (Cyldb)		

Supplementary Table 2.- Mutant neuronal and retinal phenotypes in different animal models and human listed per DUB family and gene.

ND- Not determined (The list of bibliography is included at the end)

	2dpf: small and short body, small head, necrosis spread through the body, pericardial edema, thin trunk, **notochord problem**, body bent		

Supplementary Table 2.- Mutant neuronal and retinal phenotypes in different animal models and human listed per DUB family and gene.

ND- Not determined (The list of bibliography is included at the end)

BIBLIOGRAPHY

1. Tsou W-L, Sheedlo MJ, Morrow ME, Blount JR, McGregor KM, Das C, et al. Systematic analysis of the physiological importance of deubiquitinating enzymes. PLoS One. 2012;7: e43112. doi:10.1371/journal.pone.0043112

2. Tse WKF, Eisenhaber B, Ho SHK, Ng Q, Eisenhaber F, Jiang Y-J. Genome-wide loss-of-function analysis of deubiquitylating enzymes for zebrafish development. BMC Genomics. 2009;10: 637. doi:10.1186/1471-2164-10-637

3. Ishii N, Owada Y, Yamada M, Miura S, Murata K, Asao H, et al. Loss of neurons in the hippocampus and cerebral cortex of AMSH-deficient mice. Mol Cell Biol. 2001;21: 8626–37. doi:10.1128/MCB.21.24.8626-8637.2001

4. Clague MJ, Barsukov I, Coulson JM, Liu H, Rigden DJ, Urbé S. Deubiquitylases from genes to organism. Physiol Rev. 2013;93: 1289–315. doi:10.1152/physrev.00002.2013

5. Ristic G, Tsou W-L, Todi S V. An optimal ubiquitin-proteasome pathway in the nervous system: the role of deubiquitinating enzymes. Front Mol Neurosci. 2014;7: 72. doi:10.3389/fnmol.2014.00072

6. Margolin DH, Kousi M, Chan Y-M, Lim ET, Schmahmann JD, Hadjivassiliou M, et al. Ataxia, dementia, and hypogonadotropism caused by disordered ubiquitination. N Engl J Med. 2013;368: 1992–2003. doi:10.1056/NEJMoa1215993

7. Homan CC, Kumar R, Nguyen LS, Haan E, Raymond FL, Abidi F, et al. Mutations in USP9X are associated with X-linked intellectual disability and disrupt neuronal cell migration and growth. Am J Hum Genet. Elsevier; 2014;94: 470–8. doi:10.1016/j.ajhg.2014.02.004

8. Schwickart M, Huang X, Lill JR, Liu J, Ferrando R, French DM, et al. Deubiquitinase USP9X stabilizes MCL1 and promotes tumour cell survival. Nature. 2010;463: 103–7. doi:10.1038/nature08646

9. Adorno M, Sikandar S, Mitra SS, Kuo A, Nicolis Di Robilant B, Haro-Acosta V, et al. Usp16 contributes to somatic stem-cell defects in Down's syndrome. Nature. Nature Publishing Group, a division of Macmillan Publishers Limited. All Rights Reserved.; 2013;501: 380–4. doi:10.1038/nature12530

10. Bingol B, Tea JS, Phu L, Reichelt M, Bakalarski CE, Song Q, et al. The mitochondrial deubiquitinase USP30 opposes parkin-mediated mitophagy. Nature. 2014;510: 370–5. doi:10.1038/nature13418

11. Yuasa-Kawada J, Kinoshita-Kawada M, Wu G, Rao Y, Wu JY. Midline crossing and Slit responsiveness of commissural axons require USP33. Nat Neurosci. 2009;12: 1087–1089. doi:10.1038/nn.2382

12. Tomida S, Mamiya T, Sakamaki H, Miura M, Aosaki T, Masuda M, et al. Usp46 is a quantitative trait gene regulating mouse immobile behavior in the tail suspension and forced swimming tests. Nat Genet. 2009;41: 688–695. doi:10.1038/ng.344
